# Activated cofilin exacerbates tau pathology by impairing tau-mediated microtubule dynamics

**DOI:** 10.1038/s42003-019-0359-9

**Published:** 2019-03-22

**Authors:** Jung-A. A. Woo, Tian Liu, Cenxiao C. Fang, Sara Cazzaro, Teresa Kee, Patrick LePochat, Ksenia Yrigoin, Courtney Penn, Xingyu Zhao, Xinming Wang, Stephen B. Liggett, David E. Kang

**Affiliations:** 1 USF Health Byrd Alzheimer’s Institute, University of South Florida, Morsani College of Medicine, Tampa, FL 33613 USA; 2 Department of Molecular Pharmacology and Physiology, University of South Florida, Morsani College of Medicine, Tampa, FL 33613 USA; 3 Department of Molecular Medicine, University of South Florida, Morsani College of Medicine, Tampa, FL 33613 USA; 4 James A. Haley Veteran’s Administration Hospital, Tampa, FL 33612 USA

**Keywords:** Alzheimer's disease, Mechanisms of disease

## Abstract

Alzheimer’s disease (AD) is a progressive neurodegenerative disorder and the most common form of dementia. While the accumulation of Aβ is pivotal to the etiology of AD, both the microtubule-associated protein tau (MAPT) and the F-actin severing protein cofilin are necessary for the deleterious effects of Aβ. However, the molecular link between tau and cofilin remains unclear. In this study, we found that cofilin competes with tau for direct microtubule binding in vitro, in cells, and in vivo, which inhibits tau-induced microtubule assembly. Genetic reduction of *cofilin* mitigates tauopathy and synaptic defects in Tau-P301S mice and movement deficits in tau transgenic *C. elegans*. The pathogenic effects of cofilin are selectively mediated by activated cofilin, as active but not inactive cofilin selectively interacts with tubulin, destabilizes microtubules, and promotes tauopathy. These results therefore indicate that activated cofilin plays an essential intermediary role in neurotoxic signaling that promotes tauopathy.

## Abstract (web summary)

Jung-A A. Woo et al. show that activated cofilin competes with Tau for microtubule biding in primary neuronal cultures and a *C.elegans* model. This direct binding decreases microtubule stability and promotes tauopathy, suggesting an important role for cofilin in neurotoxic signaling.

## Introduction

The two defining pathological hallmarks of Alzheimer’s disease (AD) are the accumulations of amyloid β (Aβ) and hyperphosphorylated tau, ultimately leading to synaptic and neuronal degeneration. Despite the critical importance of Aβ in the etiology of AD, multiple studies have shown that Aβ-induced neurotoxic signals require tau, as the loss of the gene coding for microtubule-associated protein tau (*MAPT*) abrogates many deleterious effects of Aβ^1–3^. However, despite the clear pathogenic link between Aβ and tau, a large knowledge gap remains in the way that Aβ pathogenically impinges on tau to promote synaptic and neuronal degeneration as well as tauopathy.

Tau is an intrinsically disordered protein that promotes tauopathy via its post-translational modification and abnormal accumulation^4–6^. The main role of tau is in the binding and stabilization of microtubules, thereby facilitating normal neuronal function^7^. Specifically, microtubule dynamics regulate neurite outgrowth and retraction as well as trafficking and transport of key proteins, vesicles, and organelles^8–14^. Axonal transport of mitochondria can be disrupted by the detachment of tau from microtubules, thereby impacting synaptic function^15–17^. Phosphorylation of tau at multiple sites, especially on Thr231, Ser262, and Ser356^18,19^, is associated with detachment of tau from microtubules. In addition to its role in microtubule assembly^20^, studies have shown that tau also associates with F-actin and promotes its bundling. In mouse and *Drosophila* models of tauopathy, tau overexpression promotes the formation of actin-rich rods^21^, aggregated structures that form under conditions of elevated ADP-actin and oxidized cofilin^22–31^. Furthermore, tau-induced F-actin bundling promotes mitochondrial dysfunction as well as oxidative stress^32^.

Another protein crucial to Aβ-induced neurotoxicity is the actin-binding protein cofilin^33^. Cofilin normally functions as a critical regulator of F-actin dynamics via its F-actin severing and depolymerizing activities. Cofilin is inactivated by phosphorylation on Ser3 by LIM kinase1 (LIMK1), whereas its dephosphorylation by Slingshot-1 (SSH1) activates cofilin^34^. Activated and intramolecular cysteine-oxidized cofilin rapidly translocates to mitochondria^35^, where it promotes mitochondrial dysfunction and apoptosis^33,36^. In addition, activated and intermolecular cysteine-oxidized cofilin together with elevated ADP-actin can form aggregates known as cofilin–actin rods^22–31^. We and others have shown that cofilin–actin pathology is widespread in AD brains^31,37^ and that cofilin activity is increased in AD brains and APP transgenic models^22,38^. Cofilin is required for Aβ-induced mitochondrial and synaptic dysfunction in primary neurons, and *cofilin* reduction also rescues defects in synaptic plasticity and contextual memory in APP/PS1 mice^22^. Despite the necessity of both tau and cofilin in Aβ-induced neurotoxicity, the molecular relationship between cofilin and tau is unclear. We hypothesized that Aβ-induced activation of cofilin represents an upstream signal that impinges on tau/microtubule regulation and tauopathy. In this study, we show that cofilin directly competes with tau for microtubule binding in vitro, in cells, and in vivo, which inhibits tau-induced microtubule assembly. Further, we show genetic and biochemical evidence directly implicating the role of “activated” cofilin in tauopathy and destabilization of tau-regulated microtubule dynamics.

## Results

### Tau–tubulin complex negatively correlates with cofilin–tubulin complex in vivo and primary neurons

**Fig. 1:**
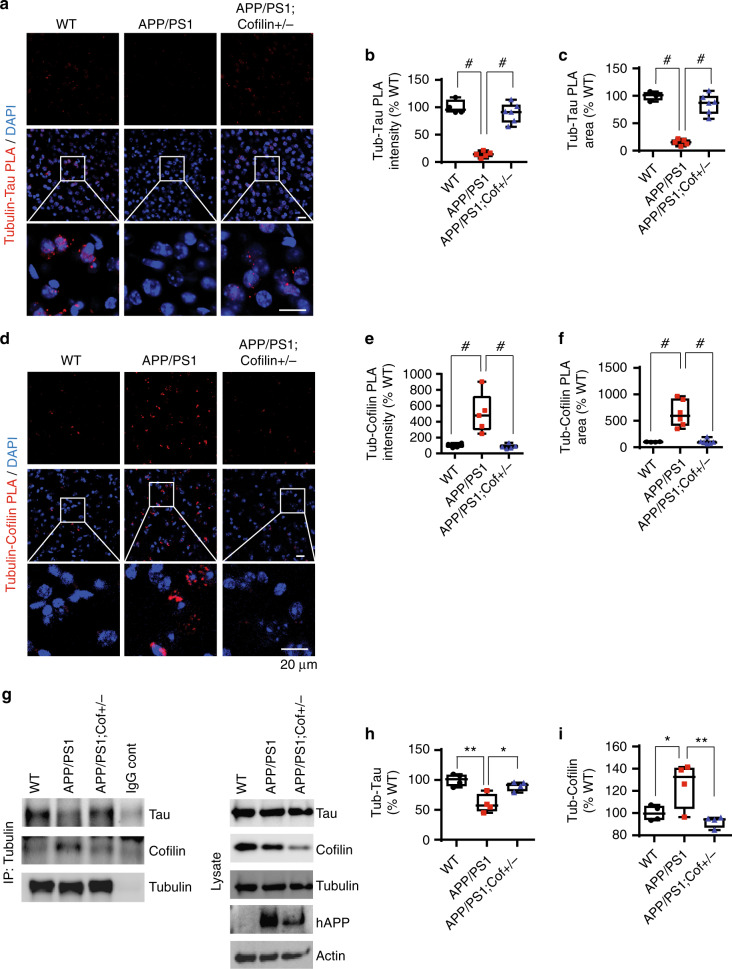
*Cofilin* reduction rescues the deficit in tau–tubulin binding in APP/PS1 mice. **a** Confocal images of tubulin–tau PLA staining in the cortex from 7-month-old WT, APP/PS1, and APP/PS1;*cofilin+/*− littermates showing significantly decreased tubulin–tau PLA signals in APP/PS1 mice (scale bar = 20 μm). **b**, **c** Quantification of tubulin–tau PLA intensity and total area. Data are expressed as mean ± SEM (one-way ANOVA with Tukey post hoc, *n* = 4/genotype, ^#^*p* < 0.0001). **d** Confocal images of tubulin–cofilin PLA staining in the cortex from 7-month-old WT, APP/PS1, and APP/PS1;*cofilin+/−* littermates showing markedly increased tubulin–cofilin PLA signals in APP/PS1 mice. mice (scale bar = 20 μm). **e**, **f** Quantification of tubulin–cofilin PLA intensity and total area. Data are expressed as mean ± SEM (one-way ANOVA with Tukey post hoc, *n* = 4/genotype, ^#^*p* < 0.0001). **g** Increased tubulin–tau complexes in APP/PS1;*cofilin+/*− compared to APP/PS1 cortical primary neurons by co-IP. **h** Quantification of tubulin–tau. Data are expressed as mean ± SEM (one-way ANOVA with Tukey post hoc, *n* = 4/genotype, *p = 0.0119, **p = 0.0018. **i** Quantification of tubulin–cofilin complexes. Data are expressed as mean ± SEM (one-way ANOVA with Tukey post hoc, *n* = 4/genotype, *p = 0.0359, **p = 0.0095)

We previously reported that Aβ42 oligomers promote cofilin activation in primary neurons and in APP/PS1 mice in vivo^39^. RNAi-mediated reduction of cofilin or SSH1 prevented Aβ42 oligomer-induced translocation of cofilin to mitochondria, mitochondrial dysfunction, synaptic protein loss, and genetic reduction of *cofilin*-abrogated deficits in long-term potentiation (LTP) in APP/PS1 mice^22^. Given multiple studies demonstrating that tau is required for Aβ-induced neurotoxicity, we hypothesized that activated cofilin could represent a key factor downstream of Aβ and upstream of tau in regulating tau–microtubule biology. Hence, we initially tested whether genetic reduction in *cofilin* impacts tau–tubulin binding in APP/PS1 mice. Utilizing the proximity ligation assay (PLA) to detect protein complexes in vivo, we observed tau–tubulin PLA puncta (Fig. 1a–c) as well as low levels of cofilin–tubulin puncta (Fig. 1d–f) in wild-type (WT) mouse brains. Neither tau–tubulin nor cofilin–tubulin PLA negative controls showed detectable signal (Supplementary Figure 1a, b). Littermate APP/PS1 brains showed a significant reduction of tau–tubulin puncta (Fig. 1a–c) together with a surprising increase in cofilin–tubulin puncta (Fig. 1d–f). However, APP/PS1;*cofilin+/*− brains significantly restored tau–tubulin complexes to levels similar to WT brains (Fig. 1a–c) together with an expected reduction in cofilin–tubulin puncta (Fig. 1d–f). To detect tubulin complexes in a different way, we performed co-immunoprecipitation (co-IP) experiments from WT, APP/PS1, and APP/PS1;*cofilin+/*− cortical primary neurons. Tau was detected in tubulin immune complexes from WT neurons, which decreased in APP/PS1 neurons (Fig. 1g, h). At the same time, cofilin was barely detectable in tubulin immune complexes from WT neurons, which significantly increased in APP/PS1 neurons (Fig. 1g, i). However, tau was restored and cofilin was diminished in tubulin immune complexes from APP/PS1;*cofilin+/−* neurons (Fig. 1g–i), indicating that *cofilin* reduction in APP/PS1 neurons restores tubulin complexes to the WT state. Even in the absence of overexpressed APP, the basal level of tau–tubulin/microtubule colocalization was significantly increased in *cofilin+/*− hippocampal primary neurons compared to littermate WT primary neurons (Supplementary Figure 1c, d). Likewise, the tau–tubulin complex was also significantly increased in *cofilin+/*− neurons compared to WT littermate neurons as detected by co-IP experiments (Supplementary Figure 1e, f), suggesting that cofilin normally negatively regulates the tau–tubulin complex.

### Cofilin directly binds tubulin, displaces tau from tubulin/microtubules, and inhibits tau-induced microtubule assembly

**Fig. 2:**
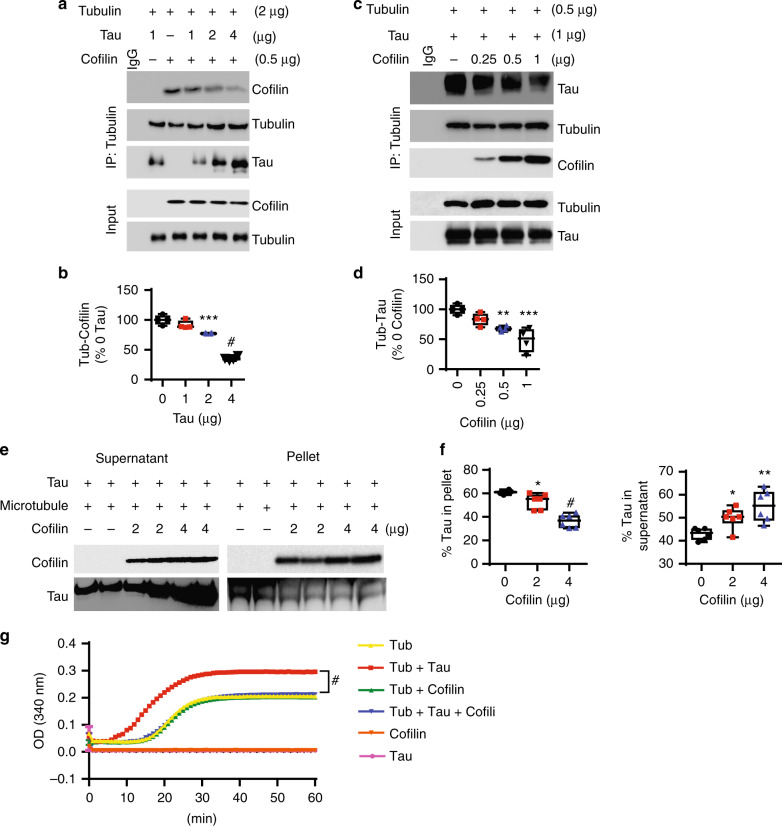
Cofilin displaces tau from tubulin/microtubules and inhibits tau-induced microtubule assembly. **a**, **b** In vitro tubulin–cofilin binding assay using recombinant proteins (His-tau, cofilin, tubulin) at indicated amounts, showing that tau reduces the tubulin–cofilin complex in a dose-dependent manner. **b** Quantification of tubulin–cofilin complexes. Data are expressed as mean ± SEM (one-way ANOVA with Tukey post hoc, *n* = 4, ***p = 0.0003, ^#^*p* < 0.0001). **c**, **d** In vitro tau–tubulin binding assay using recombinant proteins (His-tau, tubulin, and cofilin) at indicated amounts, showing that cofilin inhibits the tau–tubulin complex in a dose-dependent manner. **d** Quantification of tubulin–tau complexes. Data are expressed as mean ± SEM (one-way ANOVA with Tukey post hoc, *n* = 4, **p = 0.0073, ***p = 0.0002). **e** Binding of cofilin and tau to microtubules were performed by microtubule-binding sedimentation assay. Indicated amounts of recombinant cofilin and/or 2 µg recombinant tau were incubated with or without 0.4 nM pre-polymerized microtubules, and microtubule-associated proteins were monitored by co-sedimentation and subsequent SDS-PAGE analysis. Representative western blots showing reduced microtubule-associated tau and increased supernatant tau by cofilin in vitro. **f** Quantification of supernatant and microtubule-associated pelleted tau with indicated amounts of cofilin. Data are expressed as mean ± SEM (one-way ANOVA with Tukey post hoc, *n* = 6, supernatant tau *p = 0.0365, ^#^*p* < 0.0001, pellet tau *p = 0.0498, **p = 0.0014). **g** Tubulin polymerization was measured by turbidity at 340 nm in the presence of indicated recombinant proteins (2 μg) (two-way repeated measures ANOVA, ^#^*p* < 0.0001, *n* = 4/condition)

Based on the above in vivo and primary neuronal observations, we hypothesized that cofilin competes with tau for tubulin/microtubule binding. To determine whether cofilin can directly bind tubulin and whether such binding is competitive with tau, we first incubated a fixed amount of purified tubulin (2 μg) + cofilin (0.5 μg) with increasing amounts of purified His-tau (0, 1, 2, and 4 μg). Pull-down of tubulin demonstrated an even amount of tubulin in the immune complex (Fig. 2a). We also detected cofilin in the tubulin immune complex, which progressively diminished with increasing amounts of co-incubated tau (Fig. 2a, b), indicating that tau competitively inhibits cofilin binding to tubulin. Next, we incubated a fixed amount of purified tubulin (0.5 μg) and His-tau (1 μg) together with increasing amounts of purified cofilin (0, 0.25, 0.5, and 1 μg). As expected, pull-down of tubulin showed the presence of the tau–tubulin complex; however, the tau–tubulin complex progressively diminished with increasing amounts of co-incubated recombinant cofilin (Fig. 2c, d), indicating that cofilin competitively inhibits tau–tubulin binding. As expected, increasing amounts of BSA did not alter either tubulin–cofilin or tubulin–tau binding (Supplementary Figure 2a–d), demonstrating the specificity of these competitive protein–protein interactions. To test the binding of tau and cofilin to polymerized microtubules, we next incubated fixed amounts of recombinant His-tau with purified microtubules (RT, 30 min, 20 µM taxol) with increasing amounts of recombinant cofilin. Then the mixture was subjected to centrifugation (100,000 *g*) to separate the microtubule-bound pellet and microtubule-unbound supernatant. Similar to free tubulin, increasing amounts of cofilin significantly increased supernatant tau at the expense of tau pelleted with microtubules in a dose-dependent manner (Fig. 2e, f). At the same time, cofilin was pelleted with microtubules proportional to the reduction in pelleted tau (Fig. 2e, f). Unlike that observed with cofilin, increasing amounts of BSA neither pelleted with microtubules nor altered the amount of tau pelleted with microtubules (Supplementary Figure 2e, f), demonstrating specificity of cofilin in inhibiting the tau–microtubule interaction.

As tau is known to promote microtubule assembly in vitro^40^, we next performed in vitro microtubule assembly assays with purified tubulin, tau, and/or cofilin. In microtubule assembly assays starting with tubulin monomers, tau dramatically accelerated microtubule assembly as expected. However, purified cofilin completely prevented tau-induced acceleration of microtubule assembly, while cofilin per se had no effect on microtubule assembly (Fig. 2g). Neither tau nor cofilin alone exhibited any detectable polymerization (Fig. 2g). These results collectively support the notion that cofilin binding to tubulin/microtubules serves to competitively displace tau from microtubules, which inhibits tau-induced microtubule assembly or stability.

### Reduced *cofilin* mitigates impaired neurite outgrowth and axonal transport in Tau-P301S primary neurons

**Fig. 3:**
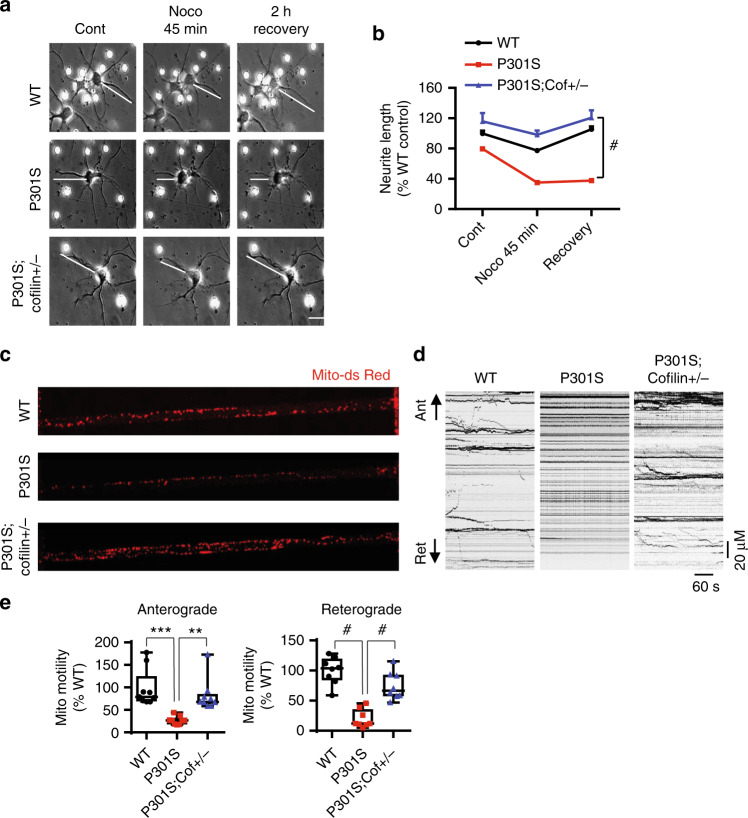
Cofilin mediates mutant tau-induced inhibition of neurite outgrowth and mitochondrial transport. **a**, **b** DIV7 hippocampal primary neurons were treated with 5 µM nocodazole for 45 min and recovered for 2 h after the removal of nocodazole. Live-cell images showing restoration of neurite length in neurons derived from WT and Tau-P301S;*cofilin+/−* neurons but not in Tau-P301S neurons after removing nocodazole (scale bar = 20 μm). **b** Quantification of neurite length of WT, Tau-P301S, and Tau-P301S;*cofilin+/−* neurons upon nocodazole treatment and after nocodazole wash-out. Data are expressed as mean ± SEM (two-way ANOVA with Bonferroni post hoc, *n* = 7–10/genotype, ^#^*p* < 0.001). **c**–**e** DIV7 cortical primary neurons derived from WT, P301S, and P301S;*cofilin+/−* were transduced with Mito-dsRed adenovirus, plated on microfluidic devices, and axonal transport was imaged in microgrooves on DIV14 by time lapse for 10 min. **d** Representative kymographs of time-lapse images of neuronal axons derived from WT, P301S, and P301S;*cofilin+/−* (scale bar = 20 μm). **e** Quantification of relative anterograde and retrograde mitochondrial motility in axons relative to WT controls. Data are expressed as mean ± SEM (one-way ANOVA with Tukey post hoc, *n* = 18–24/genotype.***p = 0.0002, **p = 0.0025, ^#^*p* < 0.0001)

Changes in microtubule dynamics are not only associated with tauopathy but are also critical for the regulation of neurite outgrowth. We treated DIV7 hippocampal primary neurons (WT, Tau-P301S, and Tau-P301S;*cofilin+/−)* with nocodazole (Fig. 3a) to depolymerize microtubules for 45 min, which showed significantly greater neurite retraction in Tau-P301S neurons compared to WT neurons as seen by live-cell imaging (Fig. 3a, b). However, neurite length in Tau-P301S;*cofilin+/−* neurons was indistinguishable from WT neurons (Fig. 3a, b). Upon recovery after nocodazole removal (2 h), Tau-P301S neurites failed to elongate further within this period, whereas both WT and Tau-P301S;*cofilin+/−* neurites fully recovered to pre-treatment levels (Fig. 3a, b).

Microtubule-based axonal transport is crucial to the delivery of vesicles and organelles (i.e. mitochondria) to presynaptic terminals. Previous studies have shown that FTD-linked mutant tau neurons (i.e. Tau-P301S) exhibit severe defects in axonal transport of mitochondria^41–43^. Therefore, we investigated whether reduced *cofilin* mitigates defects in transport of mitochondria associated with tauopathy. We transduced DIV7 primary neurons derived from WT, Tau-P301S, and Tau-P301S;*cofilin+/−* with mito-dsRed adenovirus in microfluidic devices to monitor mitochondria movement in axons (microgroove) with time-lapse live-cell imaging over a 10-min period on DIV14 (Supplementary Movies 1–3). As expected, the percentage of moving mito-dsRed particles for both anterograde and retrograde was significantly reduced in Tau-P301S versus WT neurons. However, mitochondrial movement was significantly restored in Tau-P301S;*cofilin+/−* neurons and did not differ from WT neurons (Fig. 3c–e). These results indicate that endogenous level of cofilin is required for mutant tau-induced defects in neurite outgrowth and axonal transport.

### Cofilin mediates tauopathy in Tau-P301S mice and movement impairment in tau transgenic *C. elegans*

**Fig. 4:**
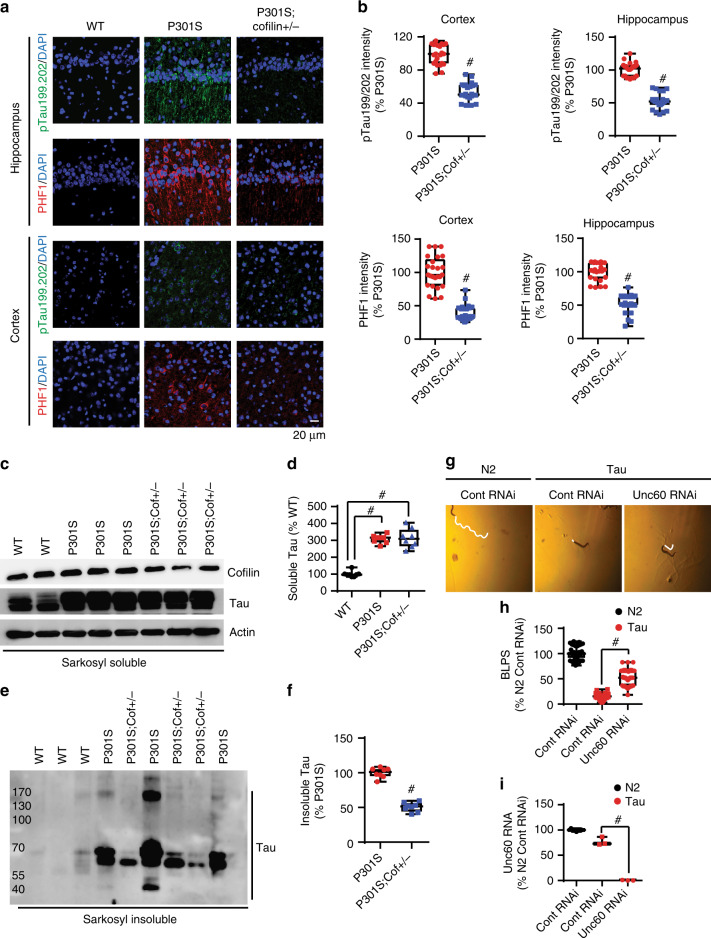
Genetic reduction of *cofilin* mitigates tauopathy in Tau-P301S mice and tau-induced movement deficits in *C. elegans*. **a**, **b** Confocal images of PHF1 (pS396/404) and phospho-tau-pS199/pS202 in the hippocampus and cortex from WT, Tau-P301S, and Tau-P301S;*cofilin+/−* littermates, showing markedly reduced PHF1 and pS199/pS202 tau in Tau-P301S;*cofilin+/−* compared to Tau-P301S (scale bar = 20 μm). **b** Quantification of intensity for PHF1 (pS396/404) and pS199/pS202 tau in Tau-P301S, and Tau-P301S;*cofilin+/−* littermates in the cortex and hippocampus. Data are expressed as mean ± SEM (*t*-test, *n* = 4/genotype, ^#^*p* < 0.0001) (scale bar = 20 μm). **c**–**f** Brain homogenates were prepared from 7-month-old WT, Tau-P301S, and Tau-P301S;*cofilin+/−* littermates. Representative immunoblots for sarkosyl-soluble and sarkosyl-insoluble tau, showing reduced sarkosyl-insoluble tau levels in Tau-P301S;*cofilin+/−* compared to Tau-P301S littermates. **d**, **f** Quantification of sarkosyl-soluble and sarkosyl-insoluble tau in WT, Tau-P301S, and Tau-P301S;*cofilin+/−* littermates. Data are expressed as mean ± SEM (one-way ANOVA with Tukey post hoc, *n* = 4/genotype, ^#^*p* < 0.0001). **g**, **h** Motility (body length per second, BLPS) on NGM plates measured at ambient room temperature (22 °C) and normalized to N2 control from 40 to 50 worms per strain (scale bar = 0.2 mm). **h** Quantification of BLPS. Data are expressed as mean ± SEM (one-way ANOVA with Tukey post hoc, *n* = 40–50 worms/condition, ^#^*p* < 0.0001). **i***C. elegans* were subjected to qRT-PCR for the *unc60* normalized to N2 control RNAi. Data are expressed as mean ± SEM (one-way ANOVA with Tukey post hoc, *n* = 40–50 worms/genotype, ^#^*p* < 0.0001)

To determine whether cofilin normally promotes tauopathy in vivo, we performed immunohistochemistry (IHC) for phospho-tau (pS396/pS404 and pS199/pS202) in the hippocampus and cortex of 7-month-old WT, Tau-P301S, and Tau-P301S;*cofilin+/−* littermates. As expected, PHF1 (pS396/pS404) and pS199/pS202 tau immunoreactivities (IRs) were starkly elevated in Tau-P301S mice compared to littermate WT mice (Fig. 4a). However, Tau-P301S;*cofilin+/−* mice exhibited significantly reduced phospho-tau IRs compared to Tau-P301S mice (Fig. 4a, b). To determine whether cofilin alters tau solubility, we subjected 7-month-old littermate mouse brains to sarkosyl soluble versus insoluble fractionation, the latter associated with tau aggregates (Fig. 4c–f). While there were no significant changes in sarkosyl-soluble tau, sarkosyl-insoluble tau was significantly decreased in Tau-P301S;*cofilin+/−* mice compared to littermate Tau-P301S mice (Fig. 4c–f). Consistent with these results in vivo, hippocampal primary neurons derived from Tau-P301S;*cofilin+/−* showed a significant >65% reduction in PHF1 IR compared to Tau-P301S neurons (Supplementary Figure 3a, b). Such changes in tauopathy were associated with a corresponding reduction in astrogliosis in Tau-P301S;*cofilin+/−* mice versus Tau-P301S mice, nearly indistinguishable from that of WT littermate mice (Supplementary Figure 4a, b).

We also tested whether reduction of cofilin improves movement in *C. elegans*-expressing WT human tau transgene (*hdEx82*). Previous studies have shown that *C. elegans*-expressing human tau exhibits movement impairment^44^. Indeed, the *hdEx82* htau *C. elegans* exhibited greatly diminished movement as expressed in body lengths per second (BLPS) compared to N2 control worms (Fig. 4g, h). Interestingly, RNAi for *unc-60*, the *C. elegans* ortholog of mammalian *cofilin*, showed a significant partial recovery of movement in *hdEx82* htau worms (Fig. 4g, h, Supplemental Movies 4–6), despite the previously reported role for *unc-60* in motility in N2 control *C. elegans*^45^. Indeed*, unc-60* mRNA was dramatically reduced in *hdEx82 C. elegans* fed with *unc-60* RNAi as detected by qRT-PCR (Fig. 4i).

### *Cofilin* reduction subverts synaptic dysfunction in Tau-P301S mice

**Fig. 5:**
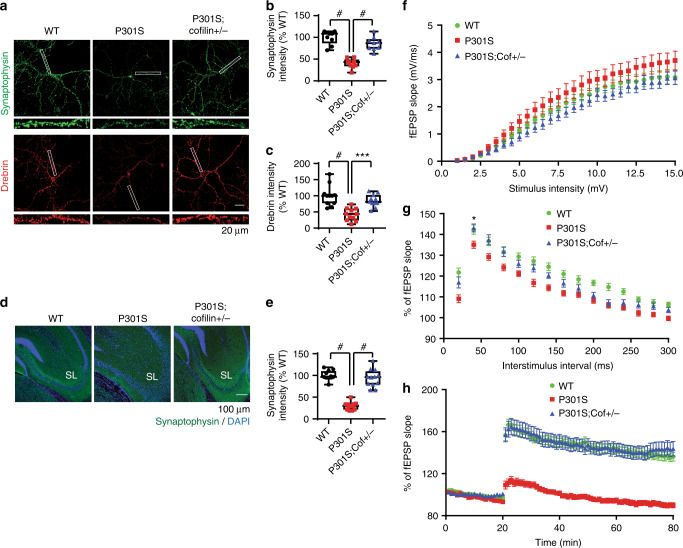
Cofilin mediates synaptic dysfunction in Tau-P301S mice. **a** Representative confocal images of synaptophysin (presynaptic, green) and drebrin (postsynaptic, red) in DIV21 hippocampal primary neurons derived from WT, Tau-P301S, and Tau-P301S;*cofilin+/−* littermates (scale bar = 20 μm). Rectangular white boxes highlight areas magnified in lower panels. **b**, **c** Quantification of synaptophysin and drebrin intensity in hippocampal primary neurons from WT, Tau-P301S, and Tau-P301S;*cofilin+/−* littermates. Data are expressed as mean ± SEM (one-way ANOVA with Tukey post hoc, *n* = 12–15/genotype, ^#^*p* < 0.0001, ***p = 0.0005). **d** Confocal images showing increased synaptophysin (green) intensity in the hippocampus of Tau-P301S;*cofilin+/−* compared to Tau-P301S littermates. **e** Quantification of synaptophysin intensity in stratum lucidum (SL). Data are expressed as mean ± SEM (one-way ANOVA with Tukey post hoc, ^#^*p* < 0.0001, *n* = 4 mice/genotype, scale bar = 100 μm). **f–h** Stimulating electrode placed in the Schaffer collaterals of the hippocampus and recording glass electrode positioned at the CA1 stratum radiatum below the pyramidal cell layer of acute slices. **f** Input/output analysis generated by stepping up stimulation amplitude from 1 to 15 mV in WT, Tau-P301S, and Tau-P301S;*cofilin+/−* acute slices. No significant differences observed. Data are expressed as mean ± SEM (WT: 30 slices, 4 mice; Tau-P301S: 31 slices, 4 mice; Tau-P301S;*cofilin+/−*: 29 slices, 4 mice). **g** PPF showing no significant differences across genotypes and interstimulus interval except between Tau-P301S;*cofilin+/−* and WT slices at the 40-ms interstimulus interval. Data are expressed as mean ± SEM (two-way ANOVA, post hoc Bonferroni, *p < 0.05; WT: 31 slices, 4 mice; Tau-P301S: 29 slices, 4 mice; Tau-P301S;*cofilin+/−*: 28 slices, 4 mice). **h** LTP induced by theta burst stimulation showing significant differences in fEPSP slope in Tau-P301S compared with WT and Tau-P301S;*cofilin+/−* slices at all time points. Data are expressed as mean ± SEM (two-way ANOVA, post hoc Bonferroni, *p* < 0.0001 at all time points, WT: 31 slices, 4 mice; Tau-P301S: 29 slices, 4 mice; Tau-P301S;*cofilin+/−*: 33 slices, 4 mice)

We next examined synaptic integrity by staining for synaptophysin and drebrin in DIV21 hippocampal neurons derived from littermate mice. As expected Tau-P301S hippocampal primary neurons showed significantly depleted drebrin and synaptophysin IRs in dendritic spines and spine-containing neurites compared to WT littermate neurons (Fig. 5a–c). However, Tau-P301S;*cofilin+/−* neurons exhibited significant recovery in both synaptophsyin and drebrin IRs, nearly indistinguishable from WT neurons (Fig. 5a–c). Likewise in 7-month-old littermate brains, synaptophysin IR was significantly reduced in stratum lucidum (SL) of Tau-P301S hippocampus compared to WT hippocampus, whereas Tau-P301S;*cofilin+/*− hippocampal SL exhibited restoration of synaptophysin IR (Fig. 5d, e).

To identify functional correlates of synaptic integrity, we examined changes in synaptic plasticity. In electrophysiological recordings of the CA3–CA1 Schaffer collateral pathway of acute brain slices, input–output (IO) analysis indicated no significant differences among WT, Tau-P301S, and Tau-P301S;*cofilin+/−* littermate slices (Fig. 5f). In paired pulse facilitation (PPF) experiments, we detected significant reductions in fEPSP slope in Tau-P301S slices in all interstimulus intervals (except 260–280 ms) compared to WT slices (Fig. 5g). However, no significant differences were detected between WT and Tau-P301S;*cofilin+/*− slices except at the 220 ms interstimulus interval (Fig. 5g), indicating that cofilin mediates Tau-P301S-induced defects in PPF. In LTP recordings using theta burst stimulation, Tau-P301S slices were dramatically impaired in the induction and maintenance of LTP compared to WT and Tau-P301;*cofilin+/−* slices. LTP in WT and Tau-P301S;*cofilin+/−* slices were indistinguishable (Fig. 5h), indicating that cofilin mediates LTP deficits in Tau-P301S mice.

### Active cofilin preferentially interacts with tubulin to disrupt microtubule dynamics

**Fig. 6:**
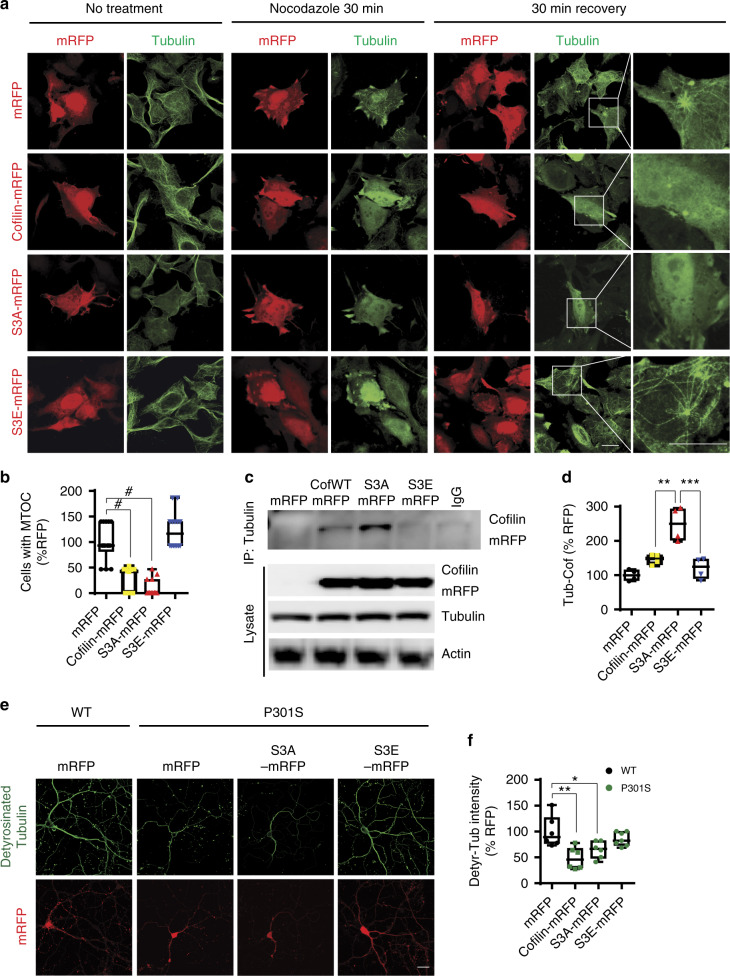
Active cofilin interacts with tubulin and disrupts microtubule dynamics. **a**, **b** Hela-V5-Tau cells were transfected with mRFP, cofilin-mRFP, cofilin-S3A-mRFP, or cofilin-S3E-mRFP for 48 h, treated with nocodazole for 30 min and recovered for another 30 min (scale bar = 10 μm). White boxes magnified to the right. **b** Quantification of transfected cells with microtubule organizing center (MTOC) normalized to mRFP controls. Data are expressed as mean ± SEM (one-way ANOVA with Tukey post hoc, *n* = 4 repeats, ^#^*p* < 0.0001). **c**, **d** Hela-V5-Tau cells were transiently transfected with control, cofilin-mRFP, cofilin-S3A-mRFP, or cofilin-S3E-mRFP mutants. Cells were lysed with 1% CHAPS buffer, and lysates were immunoprecipitated for tubulin and immunoblotted for cofilin. Representative blot shows cofilin-S3A preferentially interacts with tubulin compared to cofilin-S3E. **d** Quantification of tubulin–cofilin complexes. Data are expressed as mean ± SEM (one-way ANOVA with Tukey post hoc, *n* = 4, **p = 0.0016, ***p = 0.0003). **e**, **f** Hippocampal primary neurons derived from WT and Tau-P301S were transduced with mRFP, cofilin-mRFP, cofilin-S3A-mRFP, or cofilin-S3E-mRFP adenovirus on DIV7 and subjected to immunocytochemistry for detyrosinated tubulin on DIV21 (scale bar = 20 μm). **f** Quantification of detyrosinated tubulin intensity. Data are expressed as mean ± SEM (one-way ANOVA with Tukey post hoc, *n* = 15–20/genotype **p = 0.001, *p = 0.0228 compared to WT RFP control)

The activation and inactivation cycle of cofilin is achieved by its dephosphorylation and phosphorylation, respectively. Thus, we assessed whether a constitutively desphosphorylated (S3A, “active”) or phosphomimetic (S3E, “inactive”) cofilin differentially alters microtubule dynamics. To test this, we transfected Hela-V5-tau cells (stably expressing wild-type human tau) with monomeric red fluorescence protein (mRFP) control, cofilin-mRFP, cofilin-S3A-mRFP, or cofilin-S3E-mRFP and subjected cells to treatment with nocodazole (20 μM) for 30 min, which induces the rapid depolymerization of microtubules, after which nocodazole was washed out and cells allowed to recover for 30 min. After 30 min of nocodazole treatment, cells expressing cofilin-mRFP or cofilin-S3A-mRFP demonstrated loss of microtubules, whereas mRFP or cofilin-S3E-mRFP expressing cells still contained a visibly intact microtubule network (Fig. 6a). Even after removal of nocodazole for 30 min, little to no visible microtubule network was present in cofilin-mRFP- or cofilin-S3A-mRFP-transfected cells, whereas mRFP- or cofilin-S3E-mRFP-transfected cells demonstrated repolymerization of microtubules from the microtubule organizing center (MTOC) (Fig. 6a, b), indicating that “active” but not “inactive” cofilin disrupts microtubule polymerization. To better understand the mechanistic basis of these results, we subjected Hela-V5-Tau cells transfected with cofilin-mRFP variants to tubulin–cofilin co-IP experiments. Despite similar levels of cofilin-mRFP variant expression, significantly increased amounts of cofilin-S3A-mRFP versus cofilin-S3E-mRFP were observed in tubulin immune complexes, indicating that tubulin preferentially interacts with the active cofilin-S3A (Fig. 6c, d). To explore the impact of cofilin activation status on microtubule stability in neurons, we utilized primary neurons from WT and Tau-P301S mice. Indeed, Tau-P301S neurons exhibited significantly reduced detyrosinated tubulin, a marker of stable microtubules, compared to littermate WT neurons (Fig. 6e, f). However, transduction of cofilin-S3E-mRFP, which has previously been shown to function in a dominant negative manner^46^, restored detyrosinated tubulin to levels comparable to WT neurons (Fig. 6e, f). Interestingly, cofilin-S3A-mRFP expression in Tau-P301S neurons did not further reduce detyrosinated tubulin (Fig. 6e, f), suggesting that endogenous cofilin is sufficient to mediate Tau-P301S-induced microtubule instability.

### Active cofilin reconstitutes synaptic and tau pathology in Tau-P301S;*cofilin+/−* mice

**Fig. 7:**
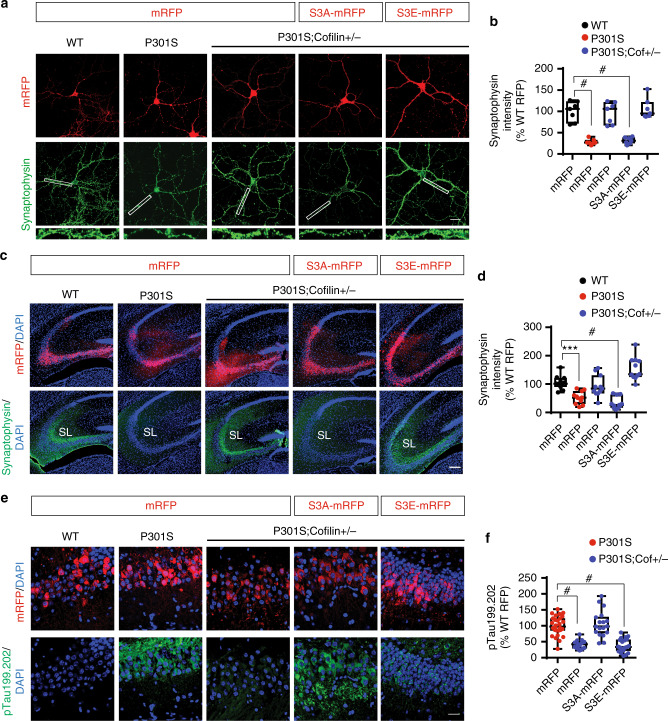
Cofilin activity is required for synaptic dysfunction and tau pathology in Tau-P301S mice. **a**, **b** Hippocampal primary neurons derived from WT, Tau-P301S, and Tau-P301S;*cofilin+/−* were transduced with mRFP, cofilin-S3A-mRFP, or cofilin-S3E-mRFP adenovirus on DIV7 and subjected to immunocytochemistry for synaptophysin on DIV21 (scale bar = 20 μm). White rectangular box areas of synaptophysin staining magnified in lower panels. **b** Quantification of synaptophysin intensity in primary neurites. Data are expressed as mean ± SEM (one-way ANOVA with Tukey post hoc, *n* = 15–20/genotype, ^#^*p* < 0.0001 compared to WT mRFP control). **c**, **d** Three-month-old WT, Tau-P301S, and Tau-P301S;*cofilin+/−* mice transduced with purified high-titer mRFP rAAV9 or cofilin-mRFP variants (S3A or S3E) rAAV9 by stereotaxic injection into the hippocampus. Brain tissues 3 months post-transduction were processed for direct confocal microscopy for mRFP and cofilin-mRFP variants as well as indirect immunohistochemistry for synaptophysin (scale bar = 100 μm). **d** Quantification of synaptophysin intensity performed from the stratum lucidum (SL) of CA3. Data are expressed as mean ± SEM (one-way ANOVA with Tukey post hoc, 4 mice/genotype, ***p = 0.0006, ^#^*p* < 0.0001 compared to WT mRFP control). **e**, **f** Three-month-old WT, Tau-P301S, and Tau-P301S;*cofilin+/−* mice transduced with purified high-titer mRFP rAAV9 or cofilin-mRFP variants (S3A or S3E) rAAV9 by stereotaxic injection into the hippocampus. Brain tissues 3 months post-transduction were processed for direct confocal microscopy for mRFP and cofilin-mRFP variants as well as indirect immunohistochemistry for tau-pS199/pS202 (scale bar = 20 μm). **f** Quantification of tau-pS199/pS202 intensity in transduced neurons. Data are expressed as mean ± SEM (one-way ANOVA with Tukey post hoc, 4 mice/genotype, ^#^*p* < 0.0001 compared to P301S mRFP control)

As *cofilin* reduction rescued tauopathy and synaptic integrity in Tau-P301S mice, we assessed whether these phenotypes can be reconstituted by expression of “active” (S3A) or “inactive” (S3E) cofilin. We first investigated synaptic integrity in transduced WT, Tau-P301S, and Tau-P301S;*cofilin+/−* primary neurons. On DIV21, Tau-P301S hippocampal primary neurons exhibited significantly decreased synaptophysin IR compared to WT and Tau-P301S;*cofilin+/−* neurons (Fig. 7a, b). In Tau-P301S;*cofilin+/−* neurons, expression of cofilin-S3A but not cofilin-S3E reconstituted the loss of synaptophysin to levels similar to Tau-P301S neurons (Fig. 7a, b).

We next assessed whether cofilin activation alters synaptic integrity and tauopathy in vivo. Hence, we generated and purified high-titer (>10^12^ vg ml^−1^) rAAV9 capable of transducing mRFP control, cofilin-S3A-mRFP, or cofilin-S3E-mRFP in brain. Purified rAAVs were then stereotaxically injected bilaterally into the hippocampus of 3-month-old WT, Tau-P301S, or Tau-P301S;*cofilin+/*− littermate mice. Three months post injection, we processed the brains for detection of mRFP, synaptic integrity, and phospho-tau levels by IHC. As expected, synaptophysin intensity within the SL of CA3 was significantly decreased in mRFP-injected Tau-P301S compared to mRFP-injected WT and Tau-P301S;*cofilin+/*− mice (Fig. 7c, d). However, synaptophysin intensity in the SL of Tau-P301S;*cofilin+/−* mice reverted to Tau-P301S levels by expression of cofilin-S3A but not cofilin-S3E (Fig. 7c, d), indicating that “active” but not “inactive” cofilin promotes Tau-P301S-induced synaptic pathology. Likewise, cofilin-S3A-mRFP but not cofilin-S3E-mRFP expression in Tau-P301S;*cofilin+/−* mice reverted phospho-tau (pS199/pS202) to Tau-P301S levels (Fig. 7e, f), indicating again that “active” but not “inactive” cofilin mediates Tau-P301S-induced tauopathy. Such changes in tauopathy secondary to expression of cofilin variants demonstrated a positive correlation with GFAP IR (astrogliosis) (Supplementary Figure 5a, b).

## Discussion

Despite the necessity of both tau and cofilin in Aβ-induced neurotoxicity, the molecular relationship between cofilin and tau is unclear. In this study, we hypothesized that Aβ-induced activation of cofilin represents an upstream signal that impinges on tau/microtubule regulation and tauopathy. We made a number of observations that shed insights to a mechanism that contributes to both Aβ-driven and mutant tau-driven tauopathy and microtubule deregulation. Specifically, we found a robust decrease in tau–microtubule complexes and increase in cofilin–microtubule complexes in APP/PS1 mice, which was reversed by reduction in cofilin, a protein activated by Aβ^22^ and increased in AD brains in an activated state^22,38^. Cofilin directly bound free tubulin and microtubules, which interfered with tau–microtubule complexes and inhibited tau-induced microtubule assembly in vitro and cell models as well as neurite outgrowth and axonal transport in primary neurons. Genetic reduction of *cofilin* robustly mitigated tauopathy and synaptic deficits in the Tau-P301S mice and movement deficits in tau transgenic *C. elegans*. Finally, we found that the “active” but not “inactive” cofilin mediates tauopathy, microtubule deregulation, and synaptic dysfunction in the Tau-P301S model, indicating that the activation cycle of cofilin represents a potential therapeutic opportunity for AD and other tauopathies.

**Fig. 8:**
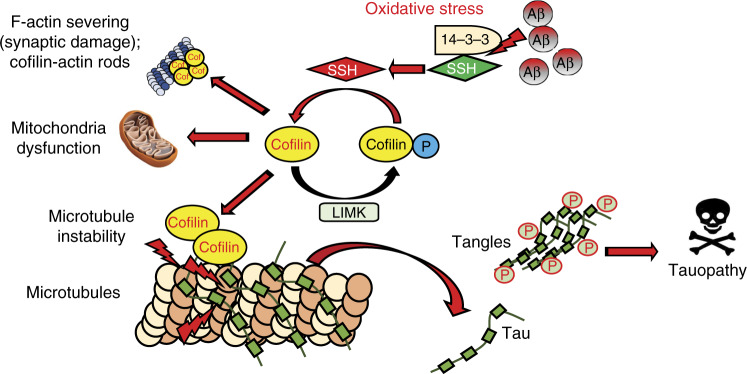
Schematic of activated cofilin in tauopathy. Under pathological conditions (i.e. Aβ and/or oxidative stress)^22,50^, oxidation of 14-3-3 releases SSH1^49^, thereby allowing cofilin activation by dephosphorylation. Activated cofilin not only deregulates F-actin dynamics (severing and cofilin–actin aggregation) and promotes mitochondrial dysfunction but also directly competes with tau for binding to microtubules. This results in displacement of tau from microtubules, destabilization of microtubules, and promotion of tauopathy

The classical function of cofilin has been ascribed to its F-actin binding and severing activity, which regulates cell motility and focal complexes^34,47,48^. This activation and deactivation cycle of cofilin phosphorylation is tightly regulated by SSH1 and LIMK1, respectively. Once activated by SSH1 and oxidized by various insults (i.e. Aβ42 oligomers), cofilin can be incorporated into cofilin–actin rods or rapidly translocates to mitochondria^22,35,49,50^ (second function), where it promotes intrinsic apoptosis in cooperation with p53^51^. In this study, we identified for the first time a surprising third function of activated cofilin—the binding to microtubules at the expense of tau and interfering with tau-induced microtubule assembly (Fig. 8). The binding of cofilin to microtubules appears to be selective for “activated” cofilin, as cofilin-S3A but not cofilin-S3E bound to tubulin. Such selectivity may be explained by the known alteration in cofilin conformation by phosphorylation/dephosphorylation^52,53^. While the function of cofilin in tau-mediated microtubule regulation may be surprising at first glance, closer examination of the literature highlights the close coordination and crosstalk between F-actin and microtubule dynamics. For example, the Rap1–cofilin pathway has been shown to regulate both F-actin and microtubule organization^54^. In *Xenopus*, cofilin has been implicated in actin filament organization, which is required for precise assembly of the MTOC^55^. While previous studies have not shown evidence of direct binding of cofilin to microtubules, a common mechanism for regulating both F-actin and microtubule dynamics as well as mitochondrial stress may be necessary for rapid coordinated adjustments in cellular processes, such as synaptic remodeling, neurite retraction, and in some cases neuronal apoptosis.

Our in vitro microtubule assembly experiments indicate that cofilin binding to tubulin per se does not interfere with microtubule assembly but only interferes with tau-induced promotion of microtubule assembly. However, we observed incomplete displacement of tau from tubulin/microtubules by cofilin in vitro but essentially complete inhibition of tau-mediated microtubule assembly by cofilin. Hence, we cannot rule out the possibility that residual cofilin binding to tau and/or tau–tubulin complexes may also render tau inactive in microtubule assembly in addition to the displacement of tau from microtubules. In Aβ-driven tauopathy (AD), we interpret our results to indicate that the chronic or excessive activation of cofilin contributes to the promotion of tauopathy. However, even in the absence of the Aβ-driven signal, it appears that endogenous level of cofilin activation is sufficient to promote tauopathy, as evidenced by the strong reduction in tauopathy in Tau-P301S mice and reconstitution by “activated” cofilin-S3A. Based on our results, we postulate that cofilin-mediated detachment of tau from microtubules may precede the hyperphosphorylation of tau in tauopathies.

Previous studies have implicated tau in F-actin bundling^21^, a process that is mechanistically distinct from F-actin polymerization and severing^21,56^. Under conditions of saturating cofilin/actin ratios, cofilin binds and saturates F-actin in a twisted form, thereby promoting the stability and bundling of F-actin rather than depolymerization. Oxidation-induced intermolecular disulfide bridging of cofilin, together with abnormal levels of ADP-actin, lead to the formation of cofilin–actin rods or aggregates (Fig. 8), which are highly enriched in brains of AD patients and APP transgenic models of AD^22–31^. However, at non-saturating activated cofilin/actin ratios, cofilin binds to ADP-actin and induces persistent F-actin severing to create new barbed and pointed ends^56^, which contributes to F-actin remodeling especially at the synapse^57^ (Fig. 8). Previous studies have also shown that Aβ oligomers promote cofilin–actin rod formations in a subset of neurons associated with NOX activation^22,24,25^ and that phospho-tau is often present together with cofilin in these structures^58,59^. These rods/aggregates, which occur in neuritic processes, have previously been shown to physically block microtubule-based axonal transport of proteins and organelles^31^. It is conceivable that activation of cofilin, increased formation of cofilin–actin rods, and sequestering of phospho-tau in some rod structures in APP/PS1 brains may in part explain the reduction in tau–tubulin complexes. If activated cofilin is capable of crosslinking F-actin and microtubules, it is also conceivable that increased active cofilin in APP/PS1 brains could increase cofilin–tubulin complexes. While such possibilities require further investigation, our data showed that cofilin can directly bind tubulin/microtubules, displace tau from microtubules, and inhibit tau-induced microtubule assembly under conditions where no actin is present. Hence, such a mechanism is one that is distinct from the action of cofilin–actin rods per se. Based on our observations of cofilin in regulating tau/microtubule dynamics, we propose that the activity of tau in F-actin bundling, which is associated with deregulation of F-actin and mitochondria^15–17^, represents an event downstream of tau detachment from microtubules at least in part induced by activated cofilin.

The findings generated from this study together with previous studies indicate that cofilin mediates both Aβ-induced neurotoxicity (i.e. via F-actin and mitochondria) and mutant tau-driven tauopathy. In both cases, activated cofilin appears to be the primary culprit. As cofilin activation is largely mediated by the phosphatase SSH1, an enzyme that is also activated by both Aβ and general oxidative stress^22,28,36^, we propose that partial inhibition of SSH1 may be a tractable approach to mitigate tauopathy (Fig. 8).

## Methods

### Mouse models

*Cofilin+/−*^22^, APP/PS1^60^, and Tau-P301S^61^ mice were maintained in the C57BL6 background for at least 10 generations and bred to generate the desired genotypes. Seven-month-old mice (male and female) were used for IHC and biochemistry experiments and 3-month-old mice (male and female) were used for electrophysiology experiments. All experiments involving mice were performed in accordance with approved protocols by the Institutional Animal Care and Use Committee (IACUC) at USF Health.

### Cell lines and primary neurons

Stably transfected Hela cells overexpressing wild-type 4R0N human tau^62^ and mouse hippocampus-derived HT22 cells^22^ have previously been described. Hippocampal and cortical primary neuron cultures were prepared from P0 pups as previously described^22^. Briefly, both hippocampus and cortex were dissected separately in ice-cold HBSS and digested with trypsin. Mouse neurons were plated on glass coverslips or plates coated with poly-d-lysine (Sigma-Aldrich, St. Louis, MO, USA) in neurobasal medium (Invitrogen, Carlsbad, CA, USA), 2% glutamax, and 2% B27 supplement (Invitrogen, Carlsbad, CA, USA).

### Antibodies, reagents, and DNA constructs

Antibodies to total tau (Tau A10, 1:1000 for Western blotting, 1:200 for cell/tissue staining; Santa Cruz Biotechnology, Dallas, TX, USA), PHF1 (kind gift from Dr. Peter Davies, 1:20, Albert Einstein College of Medicine), Tau-pS199/pS202 (1:1000 for western blotting, 1:200 for tissue staining; Invitrogen, Carlsbad, CA, USA), HT7 (1:200 for cell/tissue staining; Invitrogen, Carlsbad, CA, USA), GFAP (1:1000; Invitrogen, Carlsbad, CA, USA), synaptophysin (1:200; Invitrogen, Carlsbad, CA, USA), drebrin (1:400; Abcam, Cambridge, MA, USA), MAP2 (EMD Millipore, Billerica, MA, USA), cofilin (1:1000; Cell Signaling, Denvers, MA, USA) tubulin (1:1000; Millipore, Billerica, MA), detyrosinated tubulin (1:200; Millipore, Billerica, MA), RFP (1:200; Abcam, Cambridge, MA, USA) were used. Cofilin m-RFP adenoviral constructs and adenoviruses (WT, S3A, and S3E) were kind gifts from Dr. James Bamburg, Colorado State University (Fort Collins)^25^.

### Immunostaining

Cells were fixed with 4% paraformaldehyde for 15 min at room temperature, blocked using 3% BSA with 0.1% triton X-100 for 1 h, incubated with primary antibodies for overnight at 4% and fluorescently labeled secondary antibodies for 45 min at room temperature. For IHC, mice were perfused with PBS and fixed with 4% paraformaldehyde. Thirty-micron sections were blocked using normal goat serum for 1 h and subjected to primary antibodies at 4 °C for overnight, followed by secondary antibody (Alexa-405 and Alexa-488) incubation for 1 h at room temperature prior to mounting, as previously described^51^. Images were captured with the Olympus FV10i confocal microscope (Tokyo, Japan), and the IRs were quantified using the ImageJ software (National Institutes of Health, Bethesda, MD). IRs were quantitated from every 12th serial section through an entire hippocampus. In ICC and IHC experiments, all comparison images were acquired with identical laser intensity, exposure time, and filter. Adjustments to the brightness/contrast were applied equally to all comparison images. Regions of interest were chosen randomly, and investigators were blinded to experimental conditions during image acquisition and quantification

### Western blot analysis, immunoprecipitation, and Sarkosyl extraction

Brain homogenates and cultured cells were lysed with RIPA buffer (50 mM Tris pH 7.4, 150 mM NaCl, 2 mM ethlenediaminetetraacetic acid, 1% NP40, 0.1% sodium dodecyl sulfate) plus protease and phosphatase inhibitors. Proteins were extracted and centrifuged at 15,000 r.p.m. for 15 min at 4 ℃, and supernatants were used for western blot analysis as previously described. Uncropped blot images for all immunoblots can be found in Supplementary Figure 6 and 7. Co-immunoprecipitation (co-IP) experiments were carried out from NP40 lysates or indicated buffers with preclearing with IgG beads followed by IP with IgG beads alone or IgG beads + indicated antibodies, extensive washing (5×) with the original buffer, and western blotting on SDS-PAGE. Sarkosyl extraction was performed as previously described^63^. Briefly, brain homogenates were lysed with A68 buffer containing 10 mM Tris-HCl, pH 7.4, 0.8 M NaCl, 10% Sucrose, 1 mM EGTA. Samples were centrifuged at 400 *g* for 20 min at 4 °C, and 1% sarkosyl was added to the collected supernatants. The samples were incubated for 1.5 h and centrifuged at 80,000 *g* for 30 min at room temperature. The pellets were resuspended in 100 μl of 50 mM Tris-HCl, pH 7.4.

### PLA tissue staining

Tissue sections were washed with 0.2% triton in TBS and blocked with 0.2% Triton in 3% NGS for 1 h at room temperature. After applying primary antibodies overnight at 4 °C, tissues were washed and incubated with PLA probes at 37 °C (1 h), washed with PLA wash buffer A, incubated with ligation solution at 37 °C (30 min), washed with PLA washing buffer A, incubated with amplification solution at 37 °C (100 min), and washed with PLA washing buffer B before mounting.

### Tubulin polymerization assay

Tubulin polymerization was performed using the tubulin polymerization assay kit (Cytoskeleton Inc., Denver, CO, USA). Briefly, tubulin polymerization was monitored by measuring the absorbance readings at 340 nm. In all conditions, tubulin concentrations were 3 mg/ml in 80 mM PIPES pH 6.9, 0.5 mM EGTA, 2 mM MgCl_2_, 1 mM GTP, polymerization volumes were 100 µl. Absorbance was measured at 1 min intervals for 1 h.

### Mito-dsRed live-cell imaging

Hippocampal primary neurons were transduced with Mito-dsRed adenovirus and neurons were imaged in time lapse with the Nikon Eclipse Ti-E Fluorescence microscope (Nikon Instruments, Melville, NY, USA) at 37 °C and 5% CO_2_. Transport of mito-dsRed particles in neuronal processes was quantified with the Nikon NIS-Elements AR 3.2 software.

### Quantitative real-time RT-PCR

Quantitative real-time RT-PCR was performed using the Roche LightCycler® 96 System (Life Science, San Francisco, CA, USA). Total RNA was isolated from *C. elegans* using Trizol reagent (Invitrogen, CA, USA), reverse transcribed (Superscript III; Invitrogen, CA, USA), and subjected to quantitative PCR analysis using Syber green master mix (Invitrogen, CA, USA). The comparative threshold cycle (Ct) value was used to calculate the amplification factor, and the relative amount of unc60 was normalized to GAPDH^64^ using the following unc60 primers set: unc60-F, 5′-TGATGACTCTTCCAAGGCCG-3′; unc60-R, 5′-TCTGGGCAGATCTGGAGGAA-3′.

### Microtubule binding/sedimentation assay

Microtubule-binding assay was performed as previously described^65^. Microtubule-associated proteins were pulled down at 100,000 × *g* and subjected to SDS-PAGE.

### *C. elegans* strains and RNA interference

The *C. elegans* strains used were obtained from the Caenorhabditis Genetics Center (University of Minnesota): wild-type Bristol N2, *pha-1(e2123) III*; *hdEx82*. Strains were maintained at 25 °C to select for the Tau transgene (*hdEx82*). Strains were raised on nematode growth media (NGM) agar plates with OP50 *Escherichia coli* unless otherwise indicated. HT115 bacteria containing RNAi targeted to *unc-60* (Vidal Library, DFCIp3320H0411003D) were obtained from Source BioScience. Experimental plates were age-synchronized by bleaching. RNA interference was performed using the feeding method as previously described^66^. Briefly, age-synchronized worms were hatched on NGM places containing 1 mM IPTG and 1 mM ampicillin seeded with HT115 bacteria containing control (empty L4440 RNAi feeding vector) or RNAi targeted to *unc-60*. Worms were transferred to fresh RNAi plates every 2 days, subject to motility assays, and then harvested for mRNA quantification by qRT-PCR.

### *C. elegans* motility assay

Age-synchronized *C.elegans* per strain (8 day adults) were transferred to a fresh NGM plate. After leaving them for 30 min at RT, the *C.elegans* were recorded for 1 min at room temperature. Worm motility (BLPS) was measured using the ImageJ Plugin wrMTrck (Jesper Sondergaard Pedersen) as previously described^67^.

### Generation of rAAV9 and stereotaxic injections in mice

Recombinant AAV9 viruses were generated by co-transfection of serotype vector expressing the interest gene with pAAV9 and pXX6 in HEK293 cells and subjected to purification as previously described^68^. For brain injections, isoflurane anesthetized mice (3-month-old, equally balanced for gender per condition) were bilaterally injected with a 26-gauge needle attached to a 10-μl syringe (Hamilton, Reno, NV, USA) at the following coordinates: anteroposterior 2.7 mm, lateral 2.7 mm, and vertical 3.0 mm. A total volume of 2 μl purified rAAV9 (1 × 10^12^ vg/ml) was injected over a 2-min period using the convection enhanced delivery method. Mice were sacrificed 12 weeks post injection.

### Electrophysiology

Electrophysiological recording was performed as we previously described^22^. Briefly, hippocampus slices were prepared from 3-month-old WT, Tau-P301S, and Tau-P301S;*cofilin+/−* mice and subjected to input/output (IO) curve, PPF, and LTP recordings. The stimulating electrode was placed in the Schaffer collaterals of the hippocampus. The recording glass electrode loaded with ACSF was positioned at the CA1 stratum radiatum below the pyramidal cell layer.

### Statistical analysis and graphs

Statistical analyses were performed by the GraphPad Prism 6.0 software (GraphPad Software, San Diego, CA, USA) using Student’s *t*-test, one- or two-way ANOVA. One- or two-way ANOVA was followed by the indicated post hoc tests. All quantitative graphs with error bars were expressed as mean ± SEM.

### Reporting summary

Further information on research design is available in the Nature Research Reporting Summary linked to this article.

### Supplementary information

Supplementary Information

Descriptions of Additional Supplementary Files

Reporting Summary

Supplementary Movie 1

Supplementary Movie 2

Supplementary Movie 3

Supplementary Movie 4

Supplementary Movie 5

Supplementary Movie 6

## Data Availability

The data that support the findings of this study are available from the corresponding authors on reasonable request.

## References

[1] Morris M, Maeda S, Vossel K, Mucke L (2011). The many faces of tau. Neuron.

[2] Vossel KA (2010). Tau reduction prevents Abeta-induced defects in axonal transport. Science.

[3] Vossel KA (2015). Tau reduction prevents Abeta-induced axonal transport deficits by blocking activation of GSK3beta. J. Cell Biol..

[4] Mandelkow EM (1995). Tau domains, phosphorylation, and interactions with microtubules. Neurobiol. Aging.

[5] Mandelkow EM, Mandelkow E (2012). Biochemistry and cell biology of tau protein in neurofibrillary degeneration. Cold Spring Harb. Perspect. Med..

[6] Takalo M, Salminen A, Soininen H, Hiltunen M, Haapasalo A (2013). Protein aggregation and degradation mechanisms in neurodegenerative diseases. Am. J. Neurodegener. Dis..

[7] Drubin DG, Kirschner MW (1986). Tau protein function in living cells. J. Cell Biol..

[8] Weingarten MD, Lockwood AH, Hwo SY, Kirschner MW (1975). A protein factor essential for microtubule assembly. Proc. Natl Acad. Sci. USA.

[9] Kevenaar JT (2016). Kinesin-binding protein controls microtubule dynamics and cargo trafficking by regulating Kinesin motor activity. Curr. Biol..

[10] Delepine C (2016). Altered microtubule dynamics and vesicular transport in mouse and human MeCP2-deficient astrocytes. Hum. Mol. Genet..

[11] Heald R, Nogales E (2002). Microtubule dynamics. J. Cell Sci..

[12] Mori D (2009). An essential role of the aPKC-Aurora A-NDEL1 pathway in neurite elongation by modulation of microtubule dynamics. Nat. Cell Biol..

[13] Schaefer AW (2008). Coordination of actin filament and microtubule dynamics during neurite outgrowth. Dev. Cell.

[14] Disanza A, Scita G (2008). Cytoskeletal regulation: coordinating actin and microtubule dynamics in membrane trafficking. Curr. Biol..

[15] Reddy PH (2011). Abnormal tau, mitochondrial dysfunction, impaired axonal transport of mitochondria, and synaptic deprivation in Alzheimer’s disease. Brain Res..

[16] Lopes S (2017). Tau deletion prevents stress-induced dendritic atrophy in prefrontal cortex: role of synaptic mitochondria. Cereb. Cortex.

[17] Amadoro G (2012). Interaction between NH(2)-tau fragment and Abeta in Alzheimer’s disease mitochondria contributes to the synaptic deterioration. Neurobiol. Aging.

[18] Ando K (2016). Stabilization of microtubule-unbound Tau via Tau phosphorylation at Ser262/356 by Par-1/MARK contributes to augmentation of AD-related phosphorylation and Abeta42-induced Tau toxicity. PLoS Genet..

[19] Sengupta A (1998). Phosphorylation of tau at both Thr231 and Ser 262 is required for maximal inhibition of its binding to microtubules. Arch. Biochem. Biophys..

[20] Gustke N, Trinczek B, Biernat J, Mandelkow EM, Mandelkow E (1994). Domains of tau protein and interactions with microtubules. Biochemistry.

[21] Fulga TA (2007). Abnormal bundling and accumulation of F-actin mediates tau-induced neuronal degeneration in vivo. Nat. Cell Biol..

[22] Woo JA (2015). Slingshot-Cofilin activation mediates mitochondrial and synaptic dysfunction via Abeta ligation to beta1-integrin conformers. Cell Death Differ..

[23] PROTEIN factor essential for lactation. *Nutr. Rev*. **11**, 284–286 (1953). 10.1111/j.1753-4887.1953.tb01400.x 13087898

[24] Woo JA (2015). RanBP9 at the intersection between cofilin and Abeta pathologies: rescue of neurodegenerative changes by RanBP9 reduction. Cell Death Dis..

[25] Bamburg JR, Bernstein BW (2016). Actin dynamics and cofilin-actin rods in alzheimer disease. Cytoskeleton (Hoboken).

[26] Bamburg JR (2010). ADF/Cofilin-actin rods in neurodegenerative diseases. Curr. Alzheimer Res..

[27] Bernstein BW, Chen H, Boyle JA, Bamburg JR (2006). Formation of actin-ADF/cofilin rods transiently retards decline of mitochondrial potential and ATP in stressed neurons. Am. J. Physiol. Cell Physiol..

[28] Bernstein BW, Shaw AE, Minamide LS, Pak CW, Bamburg JR (2012). Incorporation of cofilin into rods depends on disulfide intermolecular bonds: implications for actin regulation and neurodegenerative disease. J. Neurosci..

[29] Davis RC (2009). Mapping cofilin-actin rods in stressed hippocampal slices and the role of cdc42 in amyloid-beta-induced rods. J. Alzheimers Dis..

[30] Davis RC (2011). Amyloid beta dimers/trimers potently induce cofilin-actin rods that are inhibited by maintaining cofilin-phosphorylation. Mol. Neurodegener..

[31] Minamide LS, Striegl AM, Boyle JA, Meberg PJ, Bamburg JR (2000). Neurodegenerative stimuli induce persistent ADF/cofilin-actin rods that disrupt distal neurite function. Nat. Cell Biol..

[32] DuBoff B, Gotz J, Feany MB (2012). Tau promotes neurodegeneration via DRP1 mislocalization in vivo. Neuron.

[33] Roh SE (2013). Mitochondrial dysfunction and calcium deregulation by the RanBP9-cofilin pathway. FASEB J..

[34] Bernstein BW, Bamburg JR (2010). ADF/cofilin: a functional node in cell biology. Trends Cell Biol..

[35] Chua BT (2003). Mitochondrial translocation of cofilin is an early step in apoptosis induction. Nat. Cell Biol..

[36] Klamt F (2009). Oxidant-induced apoptosis is mediated by oxidation of the actin-regulatory protein cofilin. Nat. Cell Biol..

[37] Rahman T (2014). Cofilin rods and aggregates concur with tau pathology and the development of Alzheimer’s disease. J. Alzheimers Dis..

[38] Kim T (2013). Human LilrB2 is a beta-amyloid receptor and its murine homolog PirB regulates synaptic plasticity in an Alzheimer’s model. Science.

[39] Woo JA (2012). Pivotal role of the RanBP9-cofilin pathway in Abeta-induced apoptosis and neurodegeneration. Cell Death Differ..

[40] Cleveland DW, Hwo SY, Kirschner MW (1977). Purification of tau, a microtubule-associated protein that induces assembly of microtubules from purified tubulin. J. Mol. Biol..

[41] Mellone M (2013). Tau pathology is present in vivo and develops in vitro in sensory neurons from human P301S tau transgenic mice: a system for screening drugs against tauopathies. J. Neurosci..

[42] Rodriguez-Martin T (2016). Reduced number of axonal mitochondria and tau hypophosphorylation in mouse P301L tau knockin neurons. Neurobiol. Dis..

[43] Ittner LM (2008). Parkinsonism and impaired axonal transport in a mouse model of frontotemporal dementia. Proc. Natl. Acad. Sci. USA.

[44] Brandt R, Gergou A, Wacker I, Fath T, Hutter H (2009). A Caenorhabditis elegans model of tau hyperphosphorylation: induction of developmental defects by transgenic overexpression of Alzheimer’s disease-like modified tau. Neurobiol. Aging.

[45] Ono K, Yamashiro S, Ono S (2008). Essential role of ADF/cofilin for assembly of contractile actin networks in the C. elegans somatic gonad. J. Cell Sci..

[46] Gu J (2010). ADF/cofilin-mediated actin dynamics regulate AMPA receptor trafficking during synaptic plasticity. Nat. Neurosci..

[47] Niwa R, Nagata-Ohashi K, Takeichi M, Mizuno K, Uemura T (2002). Control of actin reorganization by Slingshot, a family of phosphatases that dephosphorylate ADF/cofilin. Cell.

[48] Kurita S, Watanabe Y, Gunji E, Ohashi K, Mizuno K (2008). Molecular dissection of the mechanisms of substrate recognition and F-actin-mediated activation of cofilin-phosphatase Slingshot-1. J. Biol. Chem..

[49] Kim JS, Huang TY, Bokoch GM (2009). Reactive oxygen species regulate a slingshot-cofilin activation pathway. Mol. Biol. Cell.

[50] Walsh KP (2014). Amyloid-beta and proinflammatory cytokines utilize a prion protein-dependent pathway to activate NADPH oxidase and induce cofilin-actin rods in hippocampal neurons. PLoS. ONE.

[51] Liu T (2017). Cofilin-mediated neuronal apoptosis via p53 translocation and PLD1 regulation. Sci. Rep..

[52] Klejnot M (2013). Analysis of the human cofilin 1 structure reveals conformational changes required for actin binding. Acta Crystallogr. D Biol. Crystallogr..

[53] Nagaoka R, Abe H, Obinata T (1996). Site-directed mutagenesis of the phosphorylation site of cofilin: its role in cofilin-actin interaction and cytoplasmic localization. Cell Motil. Cytoskeleton.

[54] Wang JC (2017). The Rap1-cofilin-1 pathway coordinates actin reorganization and MTOC polarization at the B cell immune synapse. J. Cell Sci..

[55] Yamagishi Y, Abe H (2015). Reorganization of actin filaments by ADF/cofilin is involved in formation of microtubule structures during Xenopus oocyte maturation. Mol. Biol. Cell.

[56] Bamburg JR, Bernstein BW (2010). Roles of ADF/cofilin in actin polymerization and beyond. F1000 Biol. Rep..

[57] Pontrello CG (2012). Cofilin under control of beta-arrestin-2 in NMDA-dependent dendritic spine plasticity, long-term depression (LTD), and learning. Proc. Natl. Acad. Sci. USA.

[58] Whiteman IT (2009). Activated actin-depolymerizing factor/cofilin sequesters phosphorylated microtubule-associated protein during the assembly of Alzheimer-like neuritic cytoskeletal striations. J. Neurosci..

[59] Whiteman IT, Minamide LS, de Goh L, Bamburg JR, Goldsbury C (2011). Rapid changes in phospho-MAP/tau epitopes during neuronal stress: cofilin-actin rods primarily recruit microtubule binding domain epitopes. PLoS ONE.

[60] Radde R (2006). Abeta42-driven cerebral amyloidosis in transgenic mice reveals early and robust pathology. EMBO Rep..

[61] Yoshiyama Y (2007). Synapse loss and microglial activation precede tangles in a P301S tauopathy mouse model. Neuron.

[62] Jinwal UK (2009). Chemical manipulation of hsp70 ATPase activity regulates tau stability. J. Neurosci..

[63] Hasegawa M (2007). TDP-43 is deposited in the Guam parkinsonism-dementia complex brains. Brain.

[64] Liu K, Zhuang X, Mai Z (2013). p73 expression is associated with cellular chemosensitivity in human non-small cell lung cancer cell lines. Oncol. Lett..

[65] Bu W, Su LK (2001). Regulation of microtubule assembly by human EB1 family proteins. Oncogene.

[66] Woo JA (2017). Loss of function CHCHD10 mutations in cytoplasmic TDP-43 accumulation and synaptic integrity. Nat. Commun..

[67] Nussbaum-Krammer, C. I., Neto, M. F., Brielmann, R. M., Pedersen, J. S. & Morimoto, R. I. Investigating the spreading and toxicity of prion-like proteins using the metazoan model organism C. elegans. *J. Vis. Exp.* 52321, 10.3791/52321 (2015). 10.3791/52321 PMC4354510 25591151

[68] Carty N (2010). Convection-enhanced delivery and systemic mannitol increase gene product distribution of AAV vectors 5, 8, and 9 and increase gene product in the adult mouse brain. J. Neurosci. Methods.

