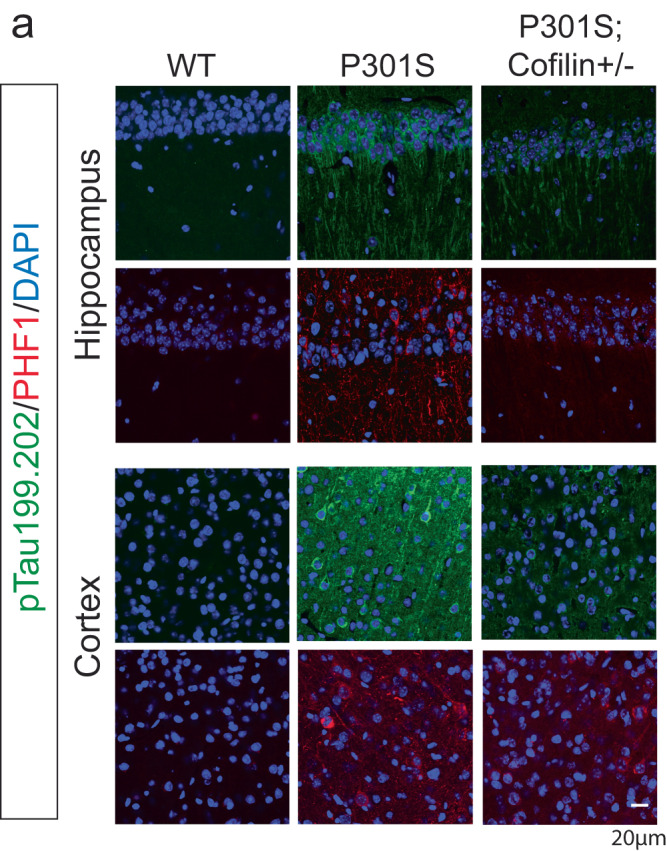# Author Correction: Activated cofilin exacerbates tau pathology by impairing tau-mediated microtubule dynamics

**DOI:** 10.1038/s42003-023-05644-x

**Published:** 2023-12-19

**Authors:** Jung-A. A. Woo, Tian Liu, Cenxiao C. Fang, Sara Cazzaro, Teresa Kee, Patrick LePochat, Ksenia Yrigoin, Courtney Penn, Xingyu Zhao, Xinming Wang, Stephen B. Liggett, David E. Kang

**Affiliations:** 1 USF Health Byrd Alzheimer’s Institute, University of South Florida, Morsani College of Medicine, Tampa, FL 33613 USA; 2 Department of Molecular Pharmacology and Physiology, University of South Florida, Morsani College of Medicine, Tampa, FL 33613 USA; 3 Department of Molecular Medicine, University of South Florida, Morsani College of Medicine, Tampa, FL 33613 USA; 4 James A. Haley Veteran’s Administration Hospital, Tampa, FL 33612 USA

Correction to: *Communications Biology* 10.1038/s42003-019-0359-9, published online 22 March 2019.

The original version of the Article contained errors in Figure 4a arising from the authors misplacing Hippocampus images for P301S and P301S;Cofilin+/- while compiling this figure.

The Figure has now been corrected.

Original Figure:
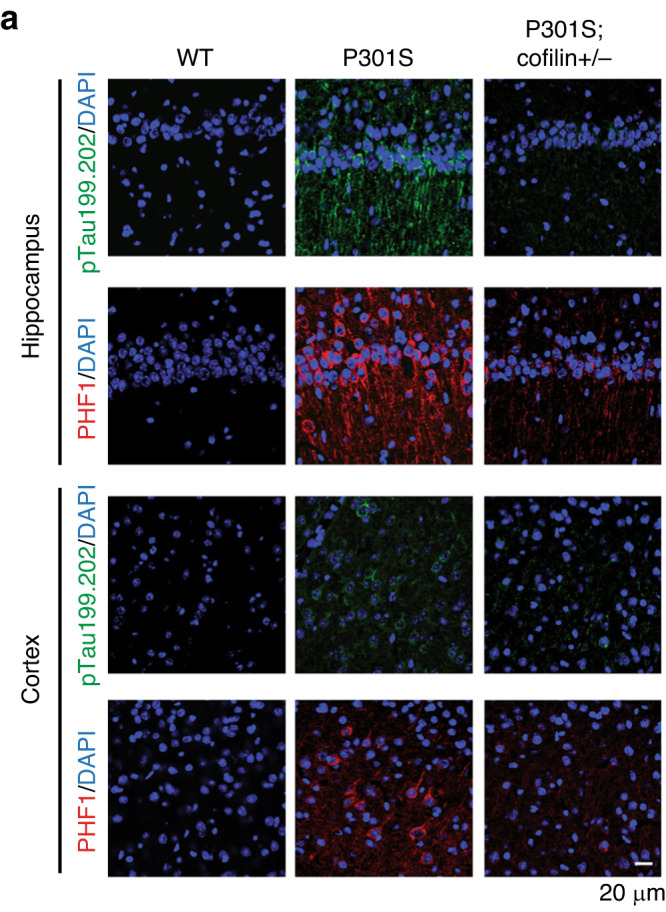


Corrected figure: