# Supplementary material for: Activated cofilin exacerbates tau pathology by impairing tau-mediated microtubule dynamics
Source: Commun Biol. 2019 Mar 22;2:112. doi: 10.1038/s42003-019-0359-9 (PMC6430779; doi:10.1038/s42003-019-0359-9)
Supplement: Supplementary file 1 — Supplementary Information [file 42003_2019_359_MOESM1_ESM.pdf]

Supplementary Figure 1

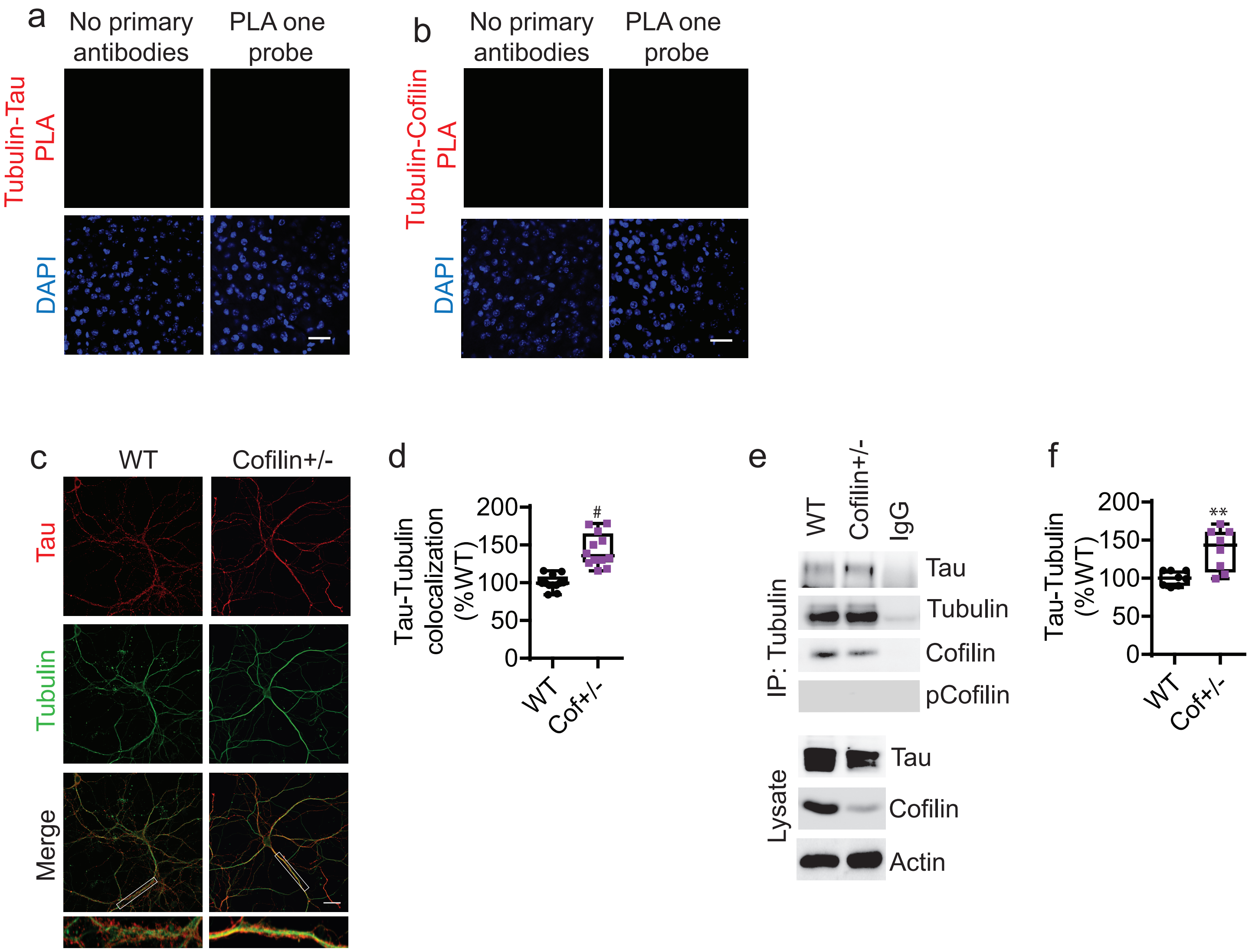

Supplementary Figure 1. Tau-tubulin complex negatively correlates with cofilin-tubulin complex. a,b Negative control confocal images of tubulin-tau and tubulin-cofilin PLA staining in cortex from 7-month old WT mice without primary antibodies (left) or with only one PLA probe (right) (scale bar = 20 µm). c,d DIV21 hippo-campal primary neurons derived from WT and cofilin+/− littermates were stained for tubulin and tau (scale bar = 20 µm). White boxed areas of merged tau and tubulin staining magnified in lower panels. d Quantification of tubulin and tau colocalization using Image J (Manders split). Data are expressed as mean ± SEM (t-test, n=19/genotype, #p<0.001). e,f Increased tubulin-tau complexes in DIV18 cortical primary neurons derived from cofilin+/− compared to WT littermates. IgG indicates negative control pull-down with IgG agarose beads. f Quantification of tubulin-tau and tubulin-cofilin complexes normalized to tau in lysates. Data are expressed as mean ± SEM (1-way ANOVA with Tukey post hoc, n=4/genotype, **p=0.0026).

Supplementary Figure 2

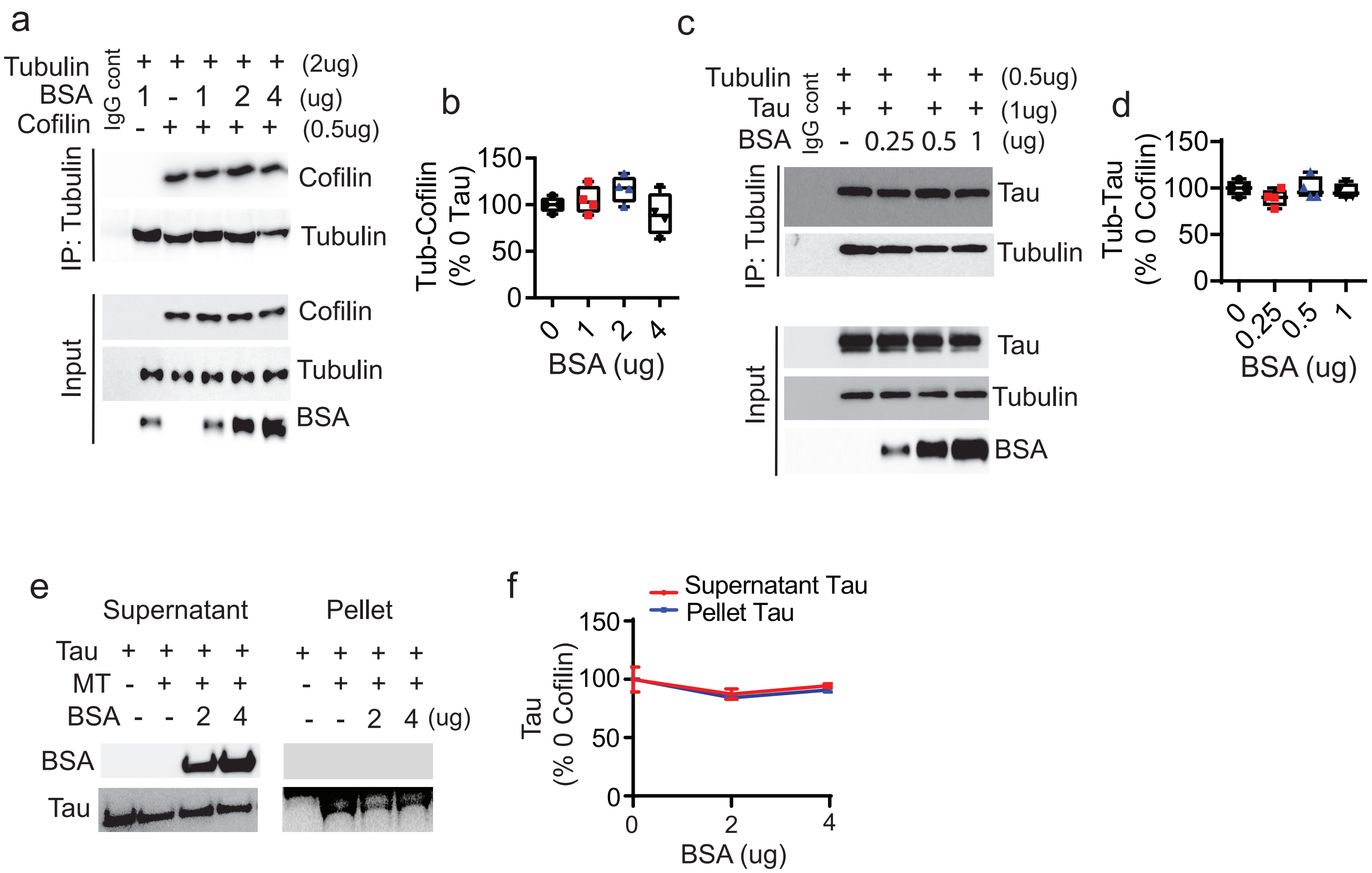

Supplementary Figure 2. Cofilin directly binds tubulin and displaces tau from tubulin/MTs. a,b Negative control experiments for in vitro tubulin-cofilin binding assay using indicated amounts of BSA, 0.5µg of cofilin, and 2µg of tubulin, showing BSA does not alter tubulin-cofilin complex. b Quantification of tubulin-cofilin complexes. Data are expressed as mean ± SEM (1-way ANOVA with Tukey post hoc, n=4). c,d Negative control experiments for in vitro tubulin-tau binding assay using indicated amounts of BSA, 0.5µg of His-tau, and 1µg of tubulin, showing BSA does not alter tubulin-tau complex. d Quantification of tubulin-tau complexes. Data are expressed as mean ± SEM (1-way ANOVA with Tukey post hoc, n=4). e,f Negative control experiments for microtubule-binding spin-down assay. Indicated amount of BSA and 2µg recombinant tau were incubated with or without 0.4 nM pre-polymerized MTs, and MT-associated proteins were monitored by co-sedimentation and subsequent SDS-PAGE analysis. f Quantification of supernatant and MT-associated pelleted tau with indicated amounts of BSA. Data are expressed as mean ± SEM (1-way ANOVA with Tukey post hoc, n=4).

Supplementary Figure 3

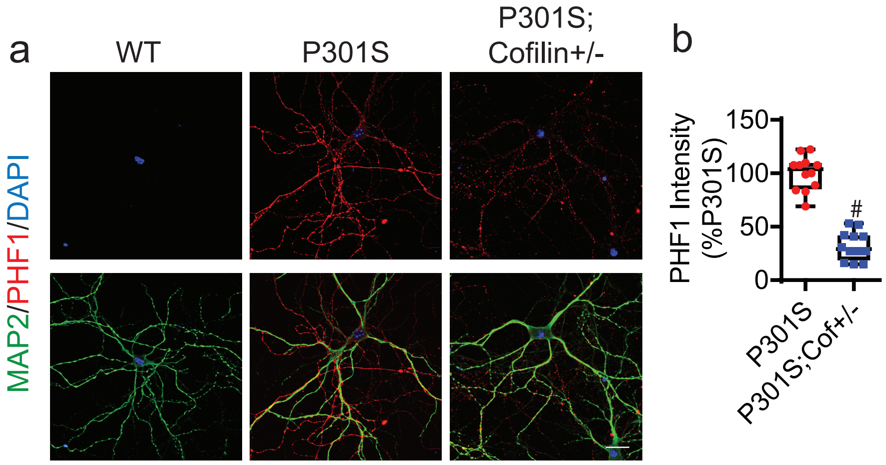

Supplementary Figure 3. Cofilin reduction decreases PHF1-tau in primary neurons. a,b Confocal images showing reduced PHF1 (red) intensity (pTau-S396/404) in Tau-P301S;cofilin+/- DIV21 hippocampal neurons compared to littermate Tau-P301S primary neurons (scale bar = 20 μm). b Quantification of PHF1 fluorescence intensity in hippocampal primary neurons. Data are expressed mean ± SEM (t-test, n=20-25/genotype, 4 mice/genotype, #p < 0.0001).

Supplementary Figure 4

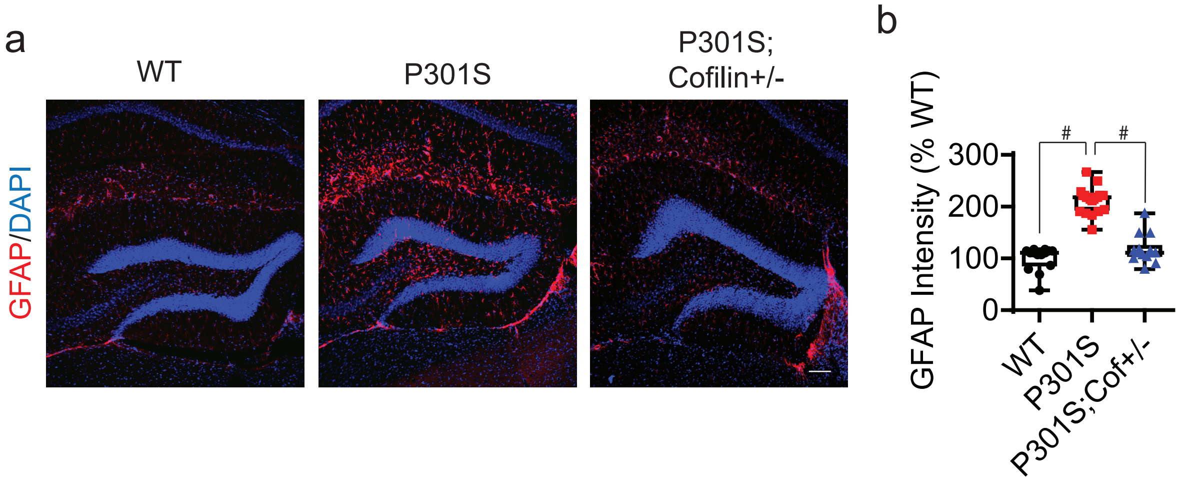

Supplementary Figure 4. Cofilin reduction mitigates astrogliosis in Tau-P301S mice. a,b Representative confocal immunofluorescence images of GFAP staining in the hippocampus of WT, Tau-P301S, and Tau-P301S;cofilin+/− littermates at 7 months of age. Stained sections were acquired with the Olympus FV10i (scale bar = 100 μm). b Quantification of GFAP intensity in the hippocampus of WT, Tau-P301S, and Tau-P301S;cofilin+/− littermates. Data are expressed as mean ± SEM (1-way ANOVA with Tukey post hoc, n=15-20/genotype, 4mice/genotype, #p<0.0001).

Supplementary Figure 5

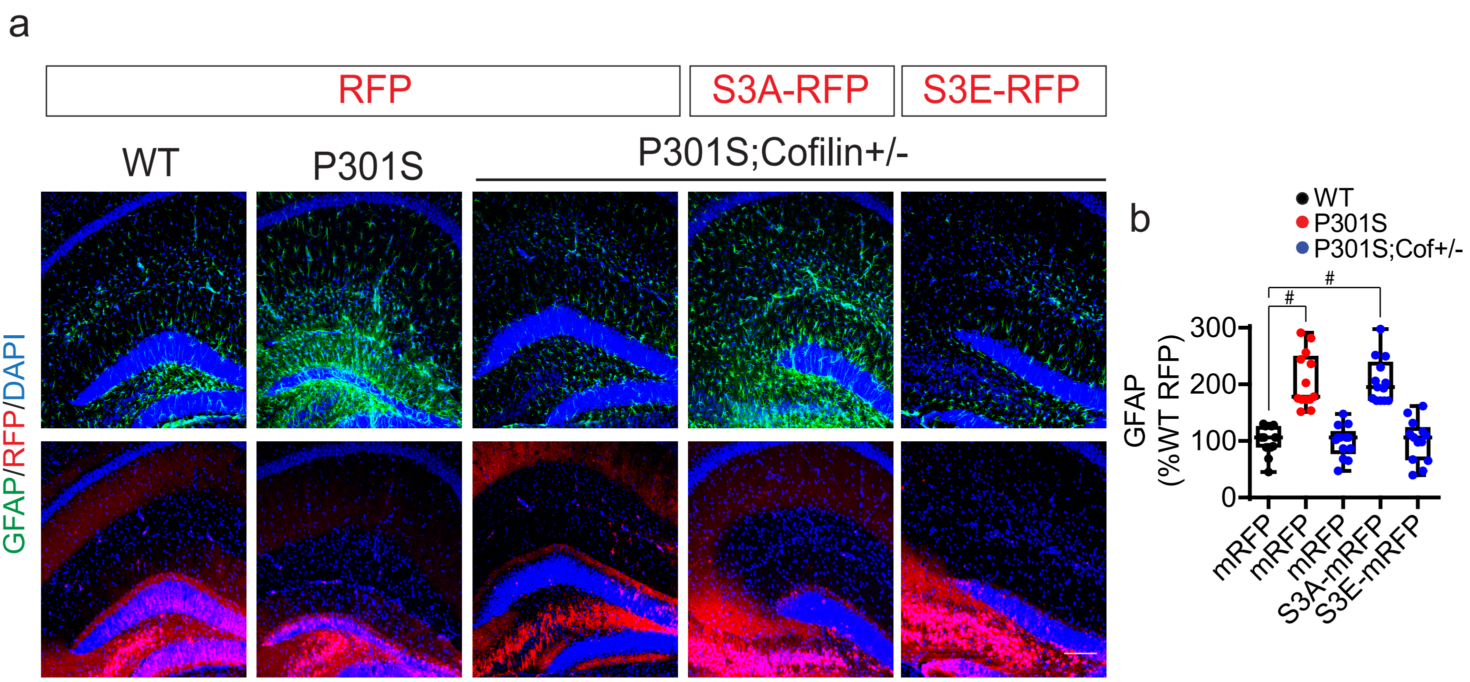

Supplementary Figure 5. Active cofilin mediates astrogliosis in Tau-P301S mice. a,b 3-month-old WT, Tau-P301S, and Tau- Tau-P301S;cofilin+/− littermate mice transduced with purified high-titer rAAV9 expressing mRFP or cofilin-mRFP variants (S3A or S3E) by stereotaxic injection into the hippocampus. Brain tissues 3-months post-injections were processed for direct confocal microscopy for mRFP and cofilin-mRFP variants as well as indirect immunohistochemistry for GFAP (scale bar = 100μm). b Quantification of GFAP intensity. Data are expressed as mean ± SEM (1-way ANOVA with Tukey post hoc, 4 mice/genotype, #p<0.0001).

Supplementary Figure 6

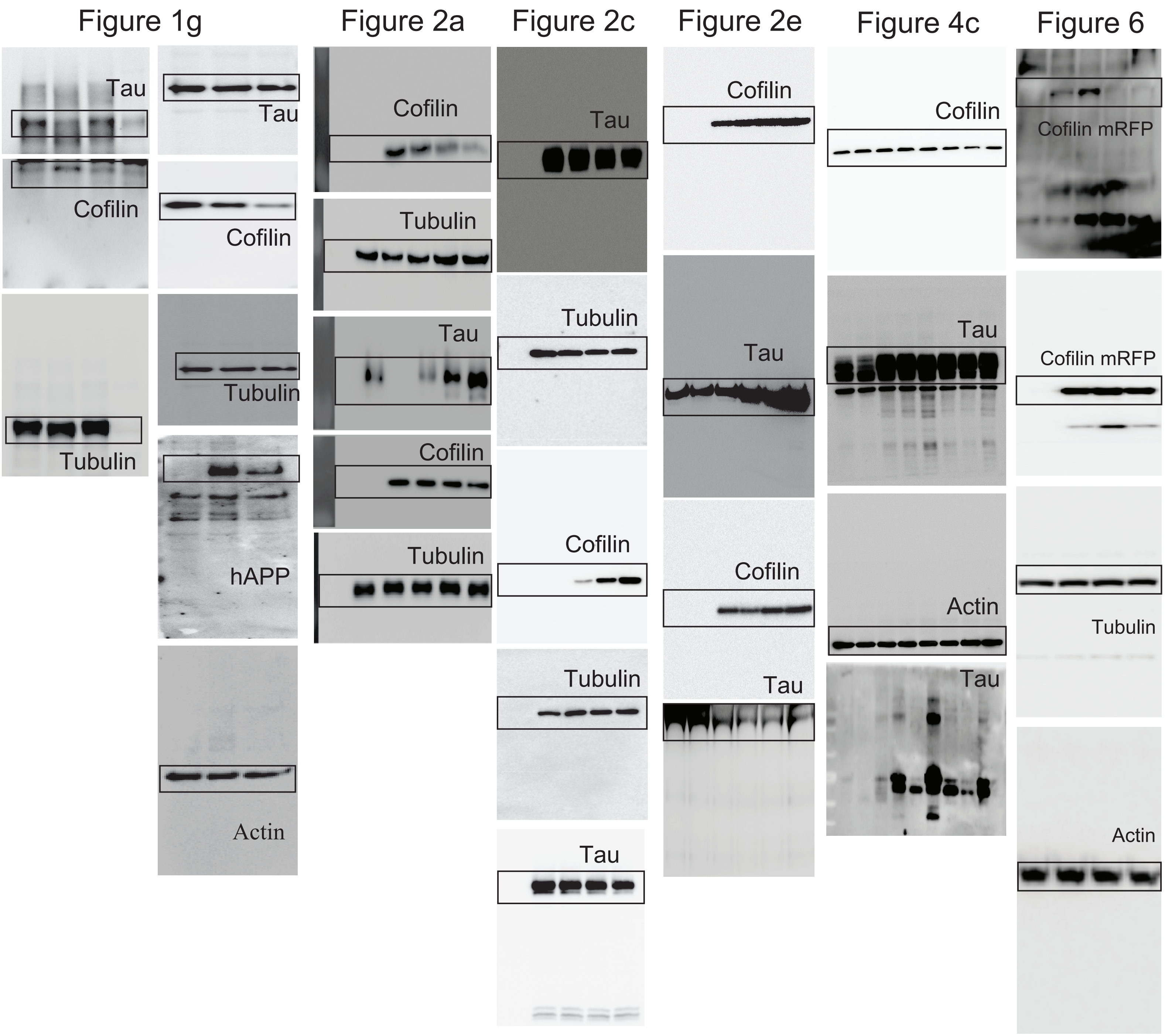

Supplementary Figure 6. Uncropped, full images of western blots

Supplementary Figure 7

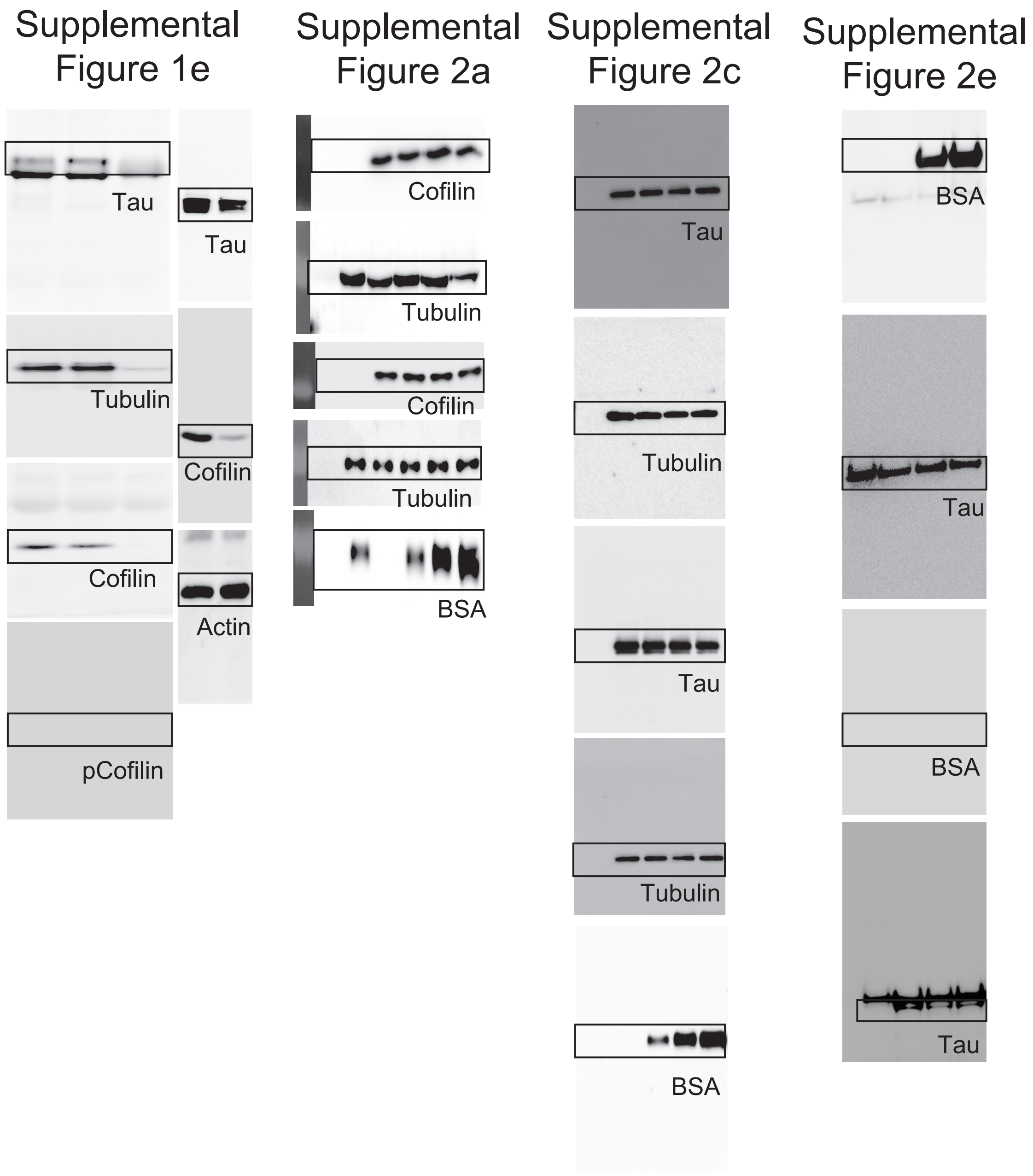

Supplementary Figure 7. Uncropped, full images of Western Blots
