# Supplementary material for: Activated cofilin exacerbates tau pathology by impairing tau-mediated microtubule dynamics
Source: Commun Biol. 2019 Mar 22;2:112. doi: 10.1038/s42003-019-0359-9 (PMC6430779; doi:10.1038/s42003-019-0359-9)
Supplement: Supplementary file 2 — Descriptions of Additional Supplementary Files [file 42003_2019_359_MOESM2_ESM.docx]

**Supplemental Movies 1-3. Reduced cofilin mitigates aberrant mitochondrial transport in Tau-P301S primary neurons. Movie 1,** Axonal mitochondrial motility in WT primary neurons. **Movie 2**, Axonal mitochondrial motility in Tau-P301S primary neurons. **Movie 3**, Axonal mitochondrial motility in Tau-P301S;*cofilin+/-* primary neurons.

**Supplemental Movies 4-6.** **Cofilin (*unc60*) knockdown partially rescues htau-induced movement deficits in *C. elegans*.** **Movie 4**, Movement of N2 control *C. elegans* fed with control RNAi. **Movie 5**, Movement of htau (*hdEx82*) *C. elegans* fed with control RNAi. **Movie 6**, Movement of htau (*hdEx82*) *C. elegans* fed with *unc60* RNAi.
