# Supplementary material for: Activated cofilin exacerbates tau pathology by impairing tau-mediated microtubule dynamics
Source: Commun Biol. 2019 Mar 22;2:112. doi: 10.1038/s42003-019-0359-9 (PMC6430779; doi:10.1038/s42003-019-0359-9)
Supplement: Supplementary file 3 — Reporting Summary [file 42003_2019_359_MOESM3_ESM.pdf]

nature research

Corresponding author(s): David E. Kang, Jung-A A. Woo

Last updated by author(s): Jan 12, 2019

Reporting Summary

Nature Research wishes to improve the reproducibility of the work that we publish. This form provides structure for consistency and transparency in reporting. For further information on Nature Research policies, see Authors & Referees and the Editorial Policy Checklist.

Statistics

For all statistical analyses, confirm that the following items are present in the figure legend, table legend, main text, or Methods section.

n/a	Confirmed	
☐	☒	The exact sample size (*n*) for each experimental group/condition, given as a discrete number and unit of measurement
☐	☒	A statement on whether measurements were taken from distinct samples or whether the same sample was measured repeatedly
☐	☒	The statistical test(s) used AND whether they are one- or two-sided<br>*Only common tests should be described solely by name; describe more complex techniques in the Methods section.*
☐	☒	A description of all covariates tested
☐	☒	A description of any assumptions or corrections, such as tests of normality and adjustment for multiple comparisons
☐	☒	A full description of the statistical parameters including central tendency (e.g. means) or other basic estimates (e.g. regression coefficient) AND variation (e.g. standard deviation) or associated estimates of uncertainty (e.g. confidence intervals)
☐	☒	For null hypothesis testing, the test statistic (e.g. F, t, r) with confidence intervals, effect sizes, degrees of freedom and P value noted<br>*Give P values as exact values whenever suitable.*
☒	☐	For Bayesian analysis, information on the choice of priors and Markov chain Monte Carlo settings
☒	☐	For hierarchical and complex designs, identification of the appropriate level for tests and full reporting of outcomes
☒	☐	Estimates of effect sizes (e.g. Cohen's *d*, Pearson's *r*), indicating how they were calculated

Our web collection on statistics for biologists contains articles on many of the points above.

Software and code

Policy information about availability of computer code

Data collection	Images were captured with the Olypus FVi confocal and Nikon Eclipse Ti-E Fluorescence microscope
Data analysis	GraphPad Prism7, Nikon NIS-Elements AR 3.2 software, and image J were used

For manuscripts utilizing custom algorithms or software that are central to the research but not yet described in published literature, software must be made available to editors/reviewers. We strongly encourage code deposition in a community repository (e.g. GitHub). See the Nature Research guidelines for submitting code & software for further information.

Data

Policy information about availability of data

All manuscripts must include a data availability statement. This statement should provide the following information, where applicable:

- Accession codes, unique identifiers, or web links for publicly available datasets
- A list of figures that have associated raw data
- A description of any restrictions on data availability

The data that support the findings of this study are available from the corresponding authors on reasonable request.

Field-specific reporting

Please select the one below that is the best fit for your research. If you are not sure, read the appropriate sections before making your selection.

☒ Life sciences ☐ Behavioural & social sciences ☐ Ecological, evolutionary & environmental sciences

For a reference copy of the document with all sections, see nature.com/documents/nr-reporting-summary-flat.pdf

Life sciences study design

All studies must disclose on these points even when the disclosure is negative.

Sample size	At least 4 mice/genotype were used for in vivo experiments.<br>At least 4 independent experiments for all the assays.
Data exclusions	No data were excluded
Replication	We replicated 3-4 times/each experiments and all confirmed that all attempts at replication were successful
Randomization	We randomly choose the samples for imaging after confirming the genotype and transfection/transduction worked
Blinding	An experimenter blind to the genotype of the mice performed in vivo experiments.

Reporting for specific materials, systems and methods

We require information from authors about some types of materials, experimental systems and methods used in many studies. Here, indicate whether each material, system or method listed is relevant to your study. If you are not sure if a list item applies to your research, read the appropriate section before selecting a response.

Materials & experimental systems

n/a	Involved in the study
☐	☒ Antibodies
☐	☒ Eukaryotic cell lines
☒	☐ Palaeontology
☐	☒ Animals and other organisms
☒	☐ Human research participants
☒	☐ Clinical data

Methods

n/a	Involved in the study
☒	☐ ChIP-seq
☒	☐ Flow cytometry
☒	☐ MRI-based neuroimaging

Antibodies

Antibodies used	Tau A10 sc-390476 Santa cruz C0217, a-tubulin ab7291 GR3197113-1 abcam, Cofilin 5175S cell signaling 4, p199.202 44-768G thermo fisher R1238605, PHF1 PF201036 invitrogen 44768G, Drebrin ab12350 abcam GR268615-8, Synaptophysin ab32127 abcam GR312544-14, Detyrosinated tubulin AB3201 millipore 2494528, MAP2 AB5622 millipore 2287690, GFAP 13-0300 thermo fisher QA213132
Validation	All primary antibodies are commercially available and validated by manufacturer, and cited by a number of published works.

Eukaryotic cell lines

Policy information about cell lines

Cell line source(s)	Hela-V5 cells, HEK293 cells
Authentication	HEK293 cells authenticated by ATCC; Hela-V5 cells authenticated by expression of hTau transgene
Mycoplasma contamination	Confirmed there was no mycoplasma contamination
Commonly misidentified lines<br>(See ICLAC register)	None

Animals and other organisms

Policy information about studies involving animals; ARRIVE guidelines recommended for reporting animal research

Laboratory animals	strain: Cofilin+/-, APP/PS1, Tau-P301S in the C57BL6 background for at least 10 generation, the same gender mice were compared, age for all experiments were stated.
Wild animals	C57BL6 background
Field-collected samples	n/a

Ethics oversight	we provided a statement of compliance with ethical regulations for the animal models

Note that full information on the approval of the study protocol must also be provided in the manuscript.
